# Yeast–*Fusarium* interactions and mycotoxin modulation in the phyllosphere

**DOI:** 10.1093/ismejo/wrag187

**Published:** 2026-07-16

**Authors:** Linda Gouka, Emma Groen, Ewa Makowicz, Jan H Christensen, Jos M Raaijmakers, Viviane Cordovez

**Affiliations:** Department of Microbial Ecology, Netherlands Institute of Ecology (NIOO-KNAW), 6708 PB, Wageningen, The Netherlands; Department of Microbial Ecology, Netherlands Institute of Ecology (NIOO-KNAW), 6708 PB, Wageningen, The Netherlands; Department of Plant and Environmental Sciences, Environmental Chemistry and Physics, University of Copenhagen, 1871, Copenhagen, Denmark; Department of Plant and Environmental Sciences, Environmental Chemistry and Physics, University of Copenhagen, 1871, Copenhagen, Denmark; Department of Microbial Ecology, Netherlands Institute of Ecology (NIOO-KNAW), 6708 PB, Wageningen, The Netherlands; Department of Molecular Biotechnology, Institute of Biology, 2333 BE, Leiden, The Netherlands; Department of Microbial Ecology, Netherlands Institute of Ecology (NIOO-KNAW), 6708 PB, Wageningen, The Netherlands

**Keywords:** yeast, phyllosphere, priority effects, Fusarium head blight, mycotoxins

## Abstract

Yeasts are prevalent members of the phyllosphere microbiome, but fundamental knowledge of the chemical basis of their interactions with other members of the phyllosphere microbiota remains largely elusive. Our previous study revealed that wheat flag leaves harbor taxonomically diverse populations of yeasts with various traits important for successful colonization and survival in the harsh phyllosphere environment. In this study, we investigated interactions between two specific yeast genera, *Aureobasidium* and *Metschnikowia*, and the mycotoxigenic fungus *Fusarium graminearum*, causal agent of Fusarium head blight (FHB). Using multiple experimental approaches, we demonstrate that phyllosphere yeasts effectively colonize wheat leaves and heads, markedly reducing FHB incidence when established before pathogen arrival. Based on metabolomic data, we further show that *Aureobasidium* and *Metschnikowia* isolates can degrade the *Fusarium* mycotoxin deoxynivalenol (DON) and DON-inducing plant compounds produced during or in response to FHB. Our findings highlight the ecology of phyllosphere yeasts and the chemical basis of their multipartite interactions with a mycotoxigenic fungus and the host plant.

## Graphical Abstract

**Graphical Abstract:**
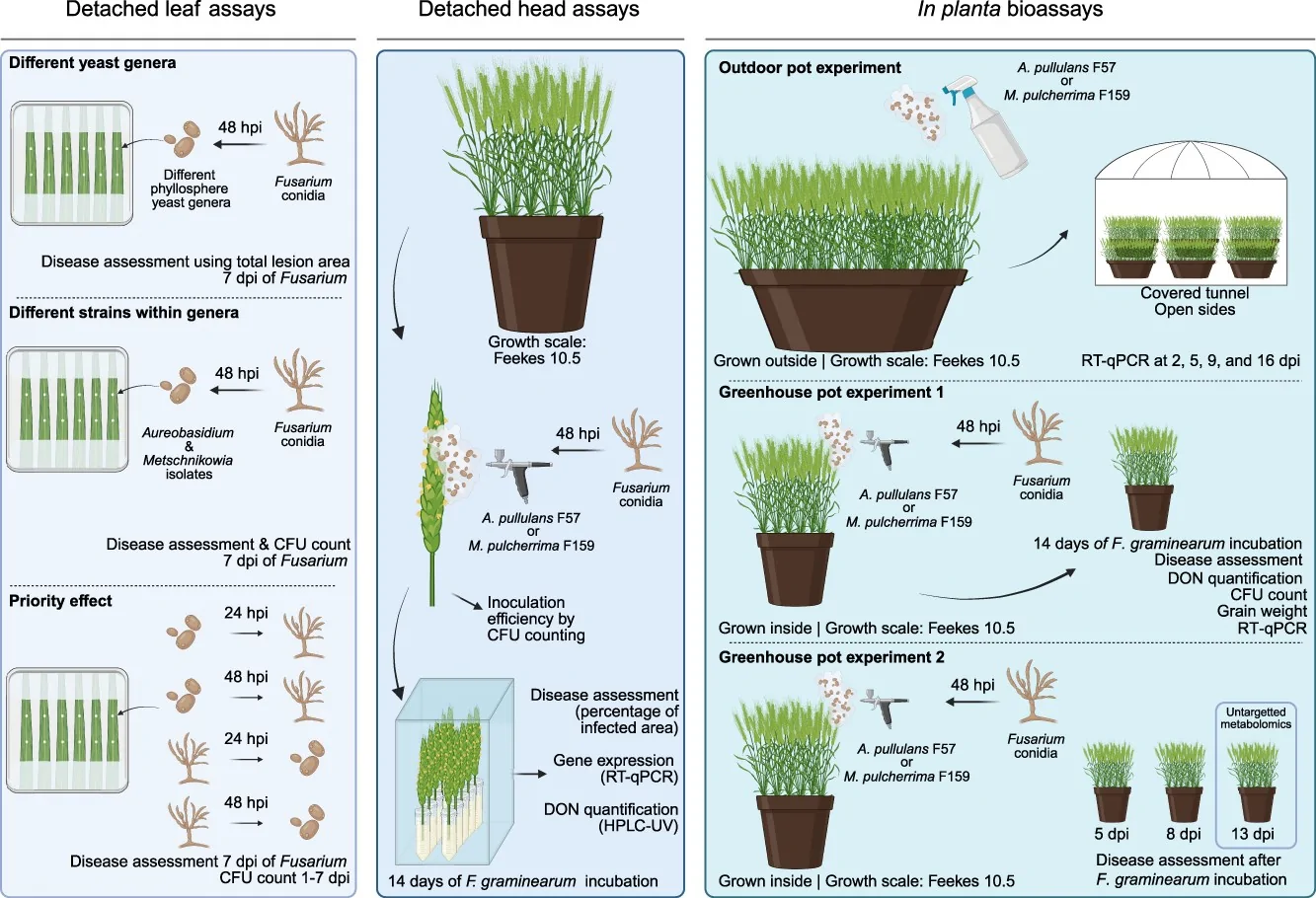

## Introduction

The phyllosphere refers to the aboveground parts of the plant and is home to a wide variety of microorganisms, including bacteria, filamentous fungi, yeasts, viruses, and archaea [1]. Among the different compartments of the phyllosphere, leaves have been the most extensively studied in microbiome research. The leaf surface is a challenging environment where microorganisms face harsh conditions, such as limited availability of nutrients, ultraviolet radiation, and fluctuating temperatures and moisture [2]. To survive and proliferate in this stressful environment, phyllosphere microorganisms employ various strategies, including metabolic adaptations and the production of extracellular polysaccharides (EPSs) and pigments [2]. Yet, the ecology of phyllosphere yeasts and the molecular and chemical basis of their interactions with other microbiota members remain poorly understood [3].

Microorganisms inhabit the leaf surface as epiphytes or colonize internal plant tissues as endophytes, resulting in complex multipartite interactions between the host plant, the epiphytic and endophytic microbiota and their surrounding environment [4]. Plants provide nutrients for these microorganisms, whereas microorganisms can confer a variety of benefits to the plant in return. For example, microbial colonization of the phyllosphere can significantly influence plant health, affecting growth, nutrient uptake, and stress tolerance to (a)biotic stresses [1–3]. Phyllosphere microorganisms can enhance plant growth through the production or modulation of hormones such as auxins, ethylene, and cytokinins [5]. Another well-studied function of plant-associated microorganisms is protection against pathogens through the induction of plant immune responses, competitive exclusion, or antibiosis, thereby hindering pathogen colonization and entry through openings in the leaf surface, such as stomata or wounds [6].

Among the phyllosphere members, bacteria represent the major leaf colonizers [4], reaching densities of 10^6^–10^8^ cells/cm^2^, frequently dominated by α- or γ-Proteobacteria, followed by β-Proteobacteria and Firmicutes [4]. The phyllosphere is also colonized by a large diversity of yeasts and filamentous fungi. Filamentous fungi inhabit both surface and internal leaf tissues, whereas yeasts primarily colonize the leaf surface at densities of 10^3^–10^5^ cells/gram [7]. Dominant yeast taxa on leaves include *Aureobasidium pullulans* [4], *Sporobolomyces* spp. [8], and *Cryptococcus* spp. [9], whereas *Metschnikowia* spp. prevail on fruit surfaces and in nectar-rich compartments of the phyllosphere [8]. Previous studies have explored the colonization dynamics of these yeasts; however, the exact cell densities are rarely reported, and can vary widely by plant host, tissue type, and geographic location [2]. For example, *A. pullulans* has been found inhabiting apple leaf surfaces at 2.0 × 10^4^ – 1.0 × 10^5^, depending on factors such as season and leaf wounding [10].

Most phyllosphere microorganisms are commensals, whereas some bacteria, filamentous fungi, and viruses act as pathogens, severely impacting crop health and yield. One of the most important (cereal) phyllosphere pathogens is *Fusarium graminearum*, the causal agent of Fusarium head blight (FHB). In addition to economic losses through shivered and empty grains, *F. graminearum* produces a spectrum of mycotoxins that threaten human and animal health [11], sometimes without visible disease symptoms [12]. Strikingly, ~60%–80% of all grains are contaminated with at least one mycotoxin type, with 25% exceeding EU legal food safety limits [13]. The most prevalent and economically damaging mycotoxin is deoxynivalenol (DON), along with its derivatives DON-3-glucoside, 3-acetyl-DON, and 15-acetyl-DON, with an incidence rate of up to 59% in cereals [13–15].

Yeasts have shown the ability to suppress fungal growth via different mechanisms, including competition for space and nutrients, direct parasitism, induction of plant resistance, and the production of antimicrobial compounds, enzymes (e.g. chitinases, glucanases), and volatiles [16]. Besides their strong antifungal activities, yeasts are phyllosphere competent [2, 16], regarded as environmentally safe and tolerant to fungicides used during post-harvest pathogen control [17]. In interactions with *F. graminearum, Cryptococcus* was able to reduce FHB in wheat heads by up to 60% but did not decrease DON content [18]. Also, *Saccharomyces cerevisiae* has been reported for its growth inhibition of *F. graminearum* and for its DON-degrading capacity [19, 20]. In this latter work, however, only *in vitro* assays were conducted and DON inhibition was most likely caused by reduced fungal growth rather than mycotoxin degradation, despite its *in vitro* potential. Our previous meta-analysis of yeast communities associated with wheat phyllosphere revealed that *S. cerevisiae* has only been reported once on wheat grains [2, 21]. This suggests that *S. cerevisiae* is likely a relatively rare and sporadic colonizer of the phyllosphere.

Yeasts are increasingly recognized as key members of plant microbiomes with potential for biocontrol, exemplified by *A. pullulans*–based formulations such as Blossom-Protect and Boni-Protect, which effectively suppress *Erwinia amylovora* (fire blight disease of apple) [16]. Although early work attributed this pathogen inhibition mainly to competitive exclusion for nutrients and space, recent multi-omics studies have also reported the modulation of host immune responses; however, no influence on pathogen biomass was observed [22]. An important ecological mechanism influencing the outcome of such microbial interactions is the priority effect, whereby the order and timing of species arrival can significantly alter community composition [23]. Early colonizers can alter nutrient availability through niche pre-emption, thereby limiting the establishment of late-arriving species. The genetic basis underlying these priority effects has been studied in nectar yeasts, including *Metschnikowia* sp., where nitrogen-scavenging genes have been implicated in fast resource acquisition and competitive dominance [24]. Together, these findings score the importance of both ecological timing and functional traits in shaping microbiome structure and biocontrol success.

Our previous work showed that the wheat phyllosphere hosts a taxonomically diverse yeast community with various traits supporting colonization and survival [25]. We also demonstrated that yeasts from different genera can inhibit *F. graminearum* hyphal growth through the production of diffusible and volatile compounds. In this study, we explored yeast–*Fusarium* interactions using detached wheat leaves and heads, as well as *in planta* bioassays. We focused on the short-term colonization dynamics of two yeast genera, *Aureobasidium* and *Metschnikowia*, their interactions with *F. graminearum*, and their ability to reduce FHB severity and DON accumulation. We also tested whether these yeasts could degrade DON and plant-derived compounds produced during or in response to FHB infection to reduce FHB disease symptoms. Our results highlight the ecology of phyllosphere yeasts and their interactions with *F. graminearum*.

## Material and methods

A graphical description of the experimental design is available as Supplementary Fig. 1.

### Yeast and fungal cultures

Yeasts were isolated from the flag leaves of wheat (*Triticum aestivum*) cultivars Elixer, Rembrandt, Kvium, Heerup, and Sheriff. Yeasts from the Elixer cultivar were previously isolated as described [25]. An additional isolation was performed for the other four cultivars, where ground flag leaf material resuspended in 15% glycerol was inoculated in Potato Dextrose Broth (PDB), and bacterial growth was prevented by adding 50 μl/ml chloramphenicol and 50 μl/ml tetracycline. Cultures were incubated at 10°C and 20°C at 180 rpm for 7–10 days before plating. Thereafter, 100 μl was plated on Potato Dextrose Agar (PDA, pH 3.5 and 5.5) and incubated for 5–7 at 10°C and 20°C. Yeast colonies were picked and streaked on fresh plates (at least twice) to obtain pure cultures. Isolate stocks were taken from plates and stored in 15% glycerol (v/v); all samples were stored at −70°C for long-term storage. Yeasts were identified based on ITS rRNA gene sequencing as described previously [25]. Taxonomy of all isolates used in this study is presented (Supplementary Table 1) [25].

For all experiments, isolates were plated on PDA (pH 5.6) and incubated at 25°C for 3–5 days. For all assays, cells were harvested by resuspension in sterile 0.9% NaCl solution and washed twice by centrifugation at 5000 rpm for 5 min. The final pellet was resuspended in 0.9% NaCl solution supplemented with 0.05% Tween20 and adjusted to the desired optical density (OD): OD_600nm_ = 0.001 for detached leaf assays, OD_600nm_ = 0.1 for outdoor pot experiments, and OD_600nm_ = 1.0 for detached head, and greenhouse pot experiments. A 100–1.000-fold lower yeast inoculum density was used on detached leaves compared to whole heads to account for difference in scale, surface area, and physiological sensitivity of the assay systems. Additionally, point inoculation was used for detached leaves, allowing targeted application and minimizing the need for high inoculum levels. In contrast, whole heads were spray-inoculated, which required higher density to ensure sufficient coverage and establishment for fungal protection.

The fungal isolate *F. graminearum* 8/1 (DSM 113711) was grown on PDA (pH 7) for 5–7 days at 25°C. For the collection of spores, three mycelial agar plugs (Ø 5 mm) were transferred to a 250 ml Erlenmeyer containing 50 ml of carboxymethyl cellulose (CMC) media (0.5 g/L MgSO_4_.7H_2_O, 1 g/L NH_4_NO_3_, 1 g/L KH_2_PO_4_, 1 g/L yeast extract, 1 g/L carboxymethyl cellulose) [26] and incubated for 7 days at 25°C while shaking (250 rpm). The obtained spore suspension was filtered through a sterile Miracloth filter (22–25 μm, Merck). Spores were washed twice with sterile 0.9% NaCl solution for 10 min at 9.500 rpm. The supernatant was discarded and the spores were resuspended in 5 ml 0.9% NaCl solution supplemented with 0.05% Tween20. A hemocytometer was used to set the conidial density at 10^5^ conidia/ml.

### Plant material and growth conditions

*Triticum aestivum* (cv. Julius) seeds were used for all in vitro and *in planta* assays (Supplementary Fig. 1). For the detached leaf assay, wheat seeds were previously sown in a seedling tray with potting soil and grown for 3 weeks in the greenhouse at 21°C (day)/17°C (night). Plates containing the detached leaves were incubated in a growth cabinet at 21°C (day)/17°C (night) under a 16-h light/8-h dark cycle. For all *in planta* assays, wheat seeds were sown in trays with potting soil and grown for 3 weeks in the greenhouse as described above. Seedlings were subsequently subjected to a cold treatment of 8 weeks at 7°C (day)/4°C (night) with 2 h light/22 h dark (70% relative humidity), to simulate winter conditions [27]. Six seedlings were transferred to a 20 L pot filled with potting soil, vermiculite (10%), and osmocote fertilizer (3 g/kg). Pots were placed in the greenhouse with 21°C (day)/17°C (night) for 16 h light/8 h dark and watered three times per week. These plants were used for detached head assays and greenhouse pot experiments 1 and 2. For the outdoor pot experiment, plants were grown outside and seeds were sown in November in pots containing potting soil, hydro clay pellets (10%), vermiculate (10%), and Osmocote fertilizer (3 g/kg). During drought periods at the end of May and beginning of June, plants were watered twice per week.

### Detached leaf assay

Detached leaf assays were performed as previously described [28] with minor modifications. Briefly, 8–10 cm leaf sections from 3-week-old wheat seedlings grown as described above were cut from the second leaf and placed with their adaxial side upwards between agar slaps containing 1.5% plant agar (pH 5.7) supplemented with 100 mg/L benzimidazole (dissolved in 96% EtOH) to delay plant senescence. To facilitate fungal infection, each leaf was slightly punctured in two different sites using sterile toothpicks.

Yeast inocula were prepared as described above, at an OD_600nm_ of 0.001 in sterile 0.9% NaCl solution supplemented with 0.05% Tween20, and a 4 μl droplet of each individual isolate (of ~100 yeast cells) was inoculated on the punctured sites of the leaf. As a control, a 4 μl droplet of 0.9% NaCl solution supplemented with 0.05% Tween20 was used. To increase humidity of the air space in the plates and to prevent dehydration of the leaves, 4 ml of sterile demi water were added to the bottom of the plates. Plates were incubated for 48 h, and after that, leaves were inoculated with a 4 μl droplet of *F. graminearum* suspension (10^5^ conidia/ml) on the punctured sites; controls were inoculated with a droplet of 0.9% NaCl solution with 0.05% Tween20. An additional 2 ml of demineralized water was added to the bottom of the plates to ensure humid conditions and plates were sealed with plastic wrap and incubated for 5–7 days before disease scoring, based on disease progression in the positive control. Two separate experiments were conducted in this study: (i) an initial screening experiment including isolates from various genera and (ii) a follow-up experiment focusing on selected *Aureobasidium* and *Metschnikowia* isolates. To test the priority effect (i.e. the influence of arrival order on community composition) either yeast isolates or *Fusarium* were inoculated first, followed by the other organism 24 or 48 h later. An overview of the yeast isolates used, their origin, and the assays in which they were included (disease scoring, yeast CFU quantification, and priority effect assays) is provided in Supplementary Table 1. Disease scoring was performed using a Leica M205C high resolution stereo microscope. Lesion size was measured by pixel counting of the diseased area via ImageJ (FIJI) after 5–7 days of incubation.

Yeast colony-forming units (CFUs) on the leaf lesions were determined at 7 days post-*Fusarium*-inoculation, while CFUs for the priority effect was determined over a 7-day period. Lesions surrounding the punctured leaf sites (0.5 cm^2^) were washed in 0.9% NaCl solution supplemented with 0.05% Tween20 by vortexing for 30 s, followed by sonication at 50 kHz for 2 min, and a second 30 s vortex. The suspension was serial diluted in 10-fold steps and plated on full-strength PDA (pH 5.6) supplemented with 50 μl/ml chloramphenicol and 50 μl/ml tetracycline to prevent bacterial growth. Yeast CFU were counted after incubation at 25°C for 2 days. The control treatment, consisting of leaves inoculated with 0.9% NaCl +0.05% Tween20, was included to assess the background microbial biomass and ensure that yeasts recovered from the experimental treatments originated from the inoculation.

### Detached head assay

Wheat heads were harvested at the flowering stage by cutting the stem around 10 cm below the head. The heads were inoculated with the yeast and the fungal isolates using an airbrush (Fengda FE-130 Airbrush Pistol, 0.3 mm nozzle). Control heads were inoculated with sterile 0.9% NaCl solution supplemented with 0.05% Tween20, and yeast-treated heads were inoculated with either *A. pullulans* F57 or *Metschnikowia pulcherrima* F159 at OD_600nm_ 1.0. Approximately 700 μl of the inoculum was evenly sprayed onto each wheat head while rotating. Inoculated wheat heads were transferred to 50 ml falcon tubes by sticking the stem into 45 ml of 1.5% plant agar supplemented with benzimidazole (100 mg/L). To determine inoculation efficiency, heads of 3 replicates per treatment were immediately transferred to 50 ml tubes containing 10 ml 0.9% NaCl solution with 0.05% Tween20. Tubes were vigorously shaken for 30 s, sonicated for 2 min and vortexed again for 30 s. After that, 10-fold serial dilutions were plated on PDA (pH 5.6, containing 50 μl/ml chloramphenicol and 50 μl/ml tetracycline) and CFU was counted after incubation at 25°C for 2 days.

Per treatment, 10 wheat heads were placed in a transparent box, previously cleaned with ethanol and UV exposure for 20 min (while opened). The boxes were sealed and placed in the greenhouse for 2 days. Afterward, the wheat heads were either inoculated with *F. graminearum* conidial suspension (10^5^/ml) or 0.9% NaCl solution with 0.05% Tween20 (control). A layer of water was added to the bottom of the transparent boxes to ensure humidity. The boxes were placed in the greenhouse and incubated for 14 days. Wheat heads were scored for FHB symptoms based on the following severity scale: 0 = no visible symptoms; 1 = 0%–25% of bleached or necrotic areas; 2 = 25%–50%; 3 = 50%–75%; 4 = 75%–99%; and 5 = 100% bleached or necrotic areas and hyphae growing outward of the wheat heads. Harvested wheat heads were transferred to clean 15 ml tubes, snap-frozen in liquid nitrogen, and stored in −8°C until mycotoxin quantification and gene expression analysis.

### FHB *in planta* assay

The *in planta* assays included an outdoor pot experiment and two greenhouse pot experiments. Plants were grown as described above, and were used for the greenhouse assay with intact wheat plants. For the outdoor pot experiment, 20 wheat seeds were sown per 90-L pot filled with a mix of potting soil, 10% vermiculite, 10% lightweight expanded clay aggregate, and 3 g/kg Osmocote fertilizer. Sowing took place in the last week of November, and pots were kept outdoors. At flowering, pots were transferred to an experimental tunnel that protected from rain while allowing airflow through open sides. In each pot, 30 wheat heads were inoculated with yeast (*A. pullulans* F57 or *M. pulcherrima* F159) at an OD_600nm_ of 0.1 at flowering (Feekes scale 10.5, mid-June). At 2, 5, 9, and 16 dpi, heads were harvested, snap-frozen in liquid nitrogen, and stored in −80°C for RNA extraction for yeast quantification.

For the greenhouse pot experiments, each pot contained 6 plants, and per pot 20 heads were inoculated upon flowering (Feekes scale 10.5) with 700 μl of 0.9% NaCl +0.05% Tween20 (negative control), *A. pullulans* F57, or *M. pulcherrima* F159 (at OD_600nm_ 1.0). After 48 h, either a conidial suspension of *F. graminearum* or 0.9% NaCl supplemented with 0.05% Tween20 (control) was inoculated onto the wheat heads as described above. The pots with the treated plants were covered by plastic foil to ensure humidity for 48 h. The experiment was repeated twice and is referred to as greenhouse pot experiment 1 and 2. For the greenhouse experiment 1, heads were harvested after 14 days, three replicates were used for CFU counting, disease scoring (40–78 heads) as described above, and snap-frozen in liquid nitrogen and stored in −80°C until mycotoxin quantification (16–20 heads) and RT-qPCR (10–11 heads). Furthermore, fresh weight measurements (20–52 heads per treatment) were sampled to determine the effect of the different treatments on ear filling. For greenhouse pot experiment 2, heads were harvested at 5, 8, and 13 days post-*F. graminearum* inoculation, disease was scored (12–15 heads) and untargeted metabolomics was performed for heads harvested at 13 days post-*F. graminearum* inoculation.

### HPLC-UV mycotoxin detection

A single wheat head, from detached head and FHB greenhouse pot experiment 1, was cut into pieces and ground manually by mortar and pestle into smaller pieces while remaining frozen (liquid nitrogen). The ground wheat head was transferred to a clean 15 ml tube containing 10 ceramic 1/4″ (6.35 mm) balls and garnet matrix (TeenPrep Lysing Matrix A, MPbio) for additional grinding. Tubes were dipped into liquid nitrogen and subjected to three rounds of automatic grinding using FastPrep24 5G (MpBio) (30 s, 6.0 m/s). To avoid any heating of the samples, the tubes were snap-frozen in liquid nitrogen in between each round of automatic grinding. After that, the ceramic beads were removed and the sample was divided over a 1.5 ml tube (~0.5 ml volume) and a 15 ml tube for RNA and DON extractions, respectively. A total of 10 replicates was prepared for the detached head assay and 16–20 replicates for the FHB greenhouse pot experiment 1.

The ground samples in 15 ml tubes were used for the extraction of the mycotoxin DON. Initially, 8 ml of ethyl acetate (with 0.1% (v/v) formic acid) was added and vortexed rigorously for 25 min. The extract was transferred to a new 15 ml tube, another 4 ml of ethyl acetate was added to the sample and subjected to another round of 25-min vortexing step. The supernatant was combined with the previously collected extract, resulting in a total volume of ~11 ml. The ethyl acetate extract was evaporated under a nitrogen gas flow and 2 ml of 99.9% methanol was added to the remaining pellet and vigorously vortexed for ~30 s to dissolve the pellet. Sample extracts were centrifuged at 21.500 rpm for 10 min, and the supernatant, containing DON was transferred to a clean 2 ml tube. Methanol was evaporated using a speedvac concentrator (Savant, SPD131DDA, manual run), and the residue was redissolved in 100 μl of 99.9% methanol for HPLC-UV analysis. Analysis was performed on a Dionex UltiMate 3000 system (ThermoFisher Scientific) consisting of a 600E pump, 2996 photodiode array detector, ZORBAX Eclipse XDB-C18 column (4.6 mm ID ×150 mm, 5 μm; Agilent Technologies). Data were processed with Chromeleon chromatography software (CDS). The mobile phase was methanol–water gradient (25:75 v/v) increasing to 100% methanol at a flow rate of 1.0 ml/min for 40 min. The column was kept at a constant temperature of 30°C and the autosampler at 18°C, and an injection volume of 2 μl was used. DON was detected at 220 nm. Quantification was based on a standard curve prepared from pure DON (Merck, The Netherlands) ranging from 1 ng/μl to 150 ng/μl. A blank sample was run after each injection to prevent carry-over and ensure thorough column cleaning. After data acquisition, each DON peak was manually inspected to assess data quality, including evaluation of retention time stability using pure DON standards (100 ng/μl) run every 10 samples.

### RNA extraction and reverse transcription quantitative PCR for fungal quantification

Single wheat heads from detached head, greenhouse pot experiment 1, and outdoor pot experiment, were ground to fine powder as described above. Fungal and wheat RNA were extracted following the protocol previously published [29] with minor modifications. Briefly, 750 μl of TRIzol were added to the 1.5 ml tube containing the ground wheat head sample. The samples were vortexed for 15 s and left to incubate at room temperature (RT) for 5 min. Afterward, samples were centrifuged for 5 min at 13.000 rpm. The resulting supernatant was transferred to 400 μl chloroform, vortexed for 15 s, and then incubated at RT for 3 min. After another round of centrifugation (15 min, 13.000 rpm, 4°C), the top layer was carefully transferred to a clean tube containing 250 μl isopropanol. Samples were kept overnight at −20°C. After that, samples were centrifuged for 15 min, 13.000 rpm at 4°C. The supernatant was discarded and the pellet was washed with 200 μl of ice-cold 75% ethanol. After air-drying for ~10 min, the pellet was resuspended in 50 μl nuclease-free water. Subsequently, the concentration and quality of the RNA (A280/260, A260/230 ratios) were measured using a nanodrop and samples were kept at −80°C.

All RT-qPCRs were performed in triplicate using a reaction volume of 10 μl. Primers were used to target housekeeping genes, which were represented by the actin gene for wheat (rrn26-F 5′-CAAATCATGTTTGAGACCTTCAATG-3′ and rrn26-R 5′-ACCAGAATCCAA-CACGATACCTG-3′ [30]) and part of the translation elongation factor 1-α for *F. graminearum* (Fa 5′-TCGTCATCGGCCACGTCGACTCT-3′ and Ra 5′-CAATGACGGTGACATAGTAGCG-3′ [31]). *TRI6* gene expression was measured with the primers TRI6-F (5′-TCTTTGTGAGCGGACGGGACTTTA-3′ and TRI6-R 5′-ATCTCGCATGTTATCCACCCTGCT-3) [32]. Previously published primers Ap3-F (5′ CTTGGTATTCCGAGGGGCA-3′) and Ap3-R (5′ CTCTCCTTTGACAGACGTTCGA-3′) [33] were used for *A. pullulans*. Specific primers Mets-F (5-GCGGCGGGAAACAAAACCAC-3′) and Mets-R (5′-GCTTGCTCACCCCGGGATTT-3′) were designed for *M. pulcherrima* using Primer3 software. A selection of nine taxonomically diverse yeast isolates from our previously established culture collection from the wheat phyllosphere were used to test the specificity of these primers [25]. These included *Cystobasidium slooffiae* (F391), *Filobasidium globisporum* (F311), *Pseudotremella moriformis* (F136), *Rhodotorula glutinis* (F224), *Holtermanniella festucosa* (F294), *Papiliotrema flavescens* (F297), *Sporobolomyces roseus* (F185), *Vishniacozyma tephrensis* (F314), and *A. pullulans* (F174) [25]. Every reaction mixture contained 5 μl iTaq Universal SYBR Green (Bio-Rad Laboratories, Hercules, CA, USA), 0.5 μl (5 pmol) of each primer, 0.125 μl of reverse transcriptase, 2.875 μl of nuclease-free water, and 1 μl of RNA sample. Except for Ap3, where 0.25 μl (0.25 pmol) of each primer, and 3.375 μl of nuclease-free water was used. Amplification consisted of an initial denaturation step of 95°C for 10 min. This was followed by 1 min at 95°C and then 40 cycles of denaturation at 95°C for 1 min. Finally, at a temperature of 63°C, annealing and extension were carried out for 30 s. RNA from wheat leaves, *A. pullulans* F57, *M. pulcherrima* F159, and *F. graminearum* mycelium were used as positive control. Cq values of FaRa, Ap, or Mets were normalized to the housekeeping gene rrn26 of wheat using the ΔCt method to account for variation in cDNA input and amplification efficiency. Relative expression was then determined by comparing ΔCt values of treated samples (inoculated wheat samples) to those of the control group (noninoculated wheat samples).

### Yeast growth in DON and other plant-derived compounds

Yeast isolates were grown in PDB supplemented with different concentrations of DON to test for toxicity. Yeast inoculums were prepared as described above and set at an OD_600nm_ of 0.01. *Aureobasidium pullulans* F57, *M. pulcherrima* F159 were grown for 48 h in PDB supplemented with 0–100 μg/ml of DON (Merck, The Netherlands) at 180 rpm in the spectrophotometer at 25°C. OD_600nm_ measurements were taken every 30 min. To evaluate the ability of the yeast isolates to grow on DON as the only carbon sources and degrade DON, these isolates were grown in minimal media (MM) [34] supplemented with 100 μg/ml of DON. Per liter, the minimal medium contained: 1.6 g of Na_2_HPO_4_,1 g of KH_2_PO_4_, 0.5 g of MgSO_4_·7H_2_O, 0.5 g of NaNO_3_, 0.5 g of (NH_4_)_2_SO_4_, 0.025 g of CaCl_2_·2H_2_O, 2 ml of trace metal solution (g/L) (1.5 g of FeCl_2_·4H_2_O, 0.190 g of CoCl_2_·6H_2_O, 0.1 g of MnCl_2_·4H_2_O, 0.07 g of ZnCl_2_, 0.062 g of H_3_BO_3_, 0.036 g of Na_2_MoO4·2H_2_O, 0.024 g of NiCl_2_·6H_2_O, 0.017 g of CuCl_2_·2H_2_O, 0.01 g of AlK(SO_4_)2·12H_2_O), and 1 ml of vitamin solution (g/L) (2 mg of biotin, 2 mg of folic acid, 5 mg of thiamine-HCl, 5 mg of riboflavin, 10 mg of pyridoxine-HCl, 50 mg of cyanocobalamin, 5 mg of niacin, 5 mg of Ca-pantothenate, 5 mg of *p*-aminobenzoate, 5 mg of thiolactic acid). A total of three replicates was prepared, and samples were taken after 9 days of incubation (25°C, 180 rpm). In addition, samples were subjected to DON extractions and HPLC-UV analysis as described. To test for degradation of other plant-derived compounds, yeast isolates were grown in MM supplemented with potent inducers of trichothecene biosynthesis: putrescine, agmatine, arginine, and guanine (Merck, the Netherlands) at 5 mM^34^. *Aureobasidium pullulans* F57, *M. pulcherrima* F159 were inoculated at an OD_600nm_ of 0.01 and incubated for 72 h with shaking at 180 rpm at 25°C. OD_600mn_ measurements were taken every 12 h with a spectrophotometer. Each experiment consisted of three biological replicates.

### Metabolome analysis of wheat heads

Metabolome analysis of wheat heads (greenhouse pot experiment 2) was performed as described in supplementary Material and Methods. Briefly, single wheat heads were ground (three replicates per treatment) using a Qiagen TissueLyser II (30 Hz, 1.5 min), and 50 mg of the homogenized powder was extracted with 1 ml of pre-cooled 80% methanol (−20°C, 3 min, 30 Hz) using two glass beads. After centrifugation (20 000 × *g*, 10 min, 4°C), extracts were analyzed on an Acquity UHPLC I-Class system coupled to a Synapt G2-S QTOF mass spectrometer (Waters) using an ACQUITY UPLC BEH C18 column (2.1 × 100 mm, 1.7 μm, 40°C). A 10-min gradient elution was performed at 0.3 ml/min with 0.1% formic acid in water (A) and methanol (B). Data were acquired in both ESI+ and ESI– modes using MSE (DIA), with leucine enkephalin as lock mass. Datafiles (.raw) were converted to .abf format and processed in MS-DIAL v4.92 for peak detection, deconvolution (MS2Dec), alignment, and drift correction (LOWESS) [35, 36]. Filtered features were further processed in MS-CleanR for clustering and feature prioritization [36]. QC-based filtering (RSD < 30%, DP ≥ 2) ensured data quality. Annotated peaks (MS-DIAL + MS-FINDER v3.60) were matched against a custom spectral library. Data visualization and normalization (log-transform, Pareto scaling) were performed in MetaboAnalyst 6.0 [37]. Raw files were deposited at https://www.ebi.ac.uk/metabolights (REQ20260309217644).

### Statistical analysis

Treatment effects were compared against FHB controls and were considered significant when *P* < .05. The Shapiro–Wilk test was used to test for normality. PERMANOVA analysis was used to indicate the influence of the experimental set-up. Student’s *t-*test or one-way ANOVA with Dunnett’s, Fisher’s, or Tukey HSD correction were used for normally distributed data, Mann–Whitney *U* test, and Kruskal–Wallis with Bonferroni correction and Dunn’s multiple comparisons method were used when non-normally distributed treatments were compared to the control (*Fusarium*-inoculated) sample.

## Results

### *Aureobasidium* and *Metschnikowia* proliferate on wheat leaves and reduce *Fusarium* infection

We previously showed that taxonomically diverse yeasts isolated from wheat flag leaves inhibit *F. graminearum* hyphal growth and suppress leaf infections under controlled laboratory (*in vitro*) conditions [25]. To assess the potential of these isolates to inhibit the fungal pathogen in the phyllosphere, we used a detached leaf assay. Lesion-containing leaf sections from each Petri dish (each containing five to seven leaves) were pooled and treated as a single replicate, resulting in three to five replicates per treatment (Supplementary Fig. 2, Supplementary Table 1). *Aureobasidium* and *Metschnikowia* isolates significantly and consistently suppressed hyphal growth and leaf infections (linear mixed-effects model, Dunnett’s correction, *P* < .05) and were thus selected for mechanistic analyses.

**Figure 1:**
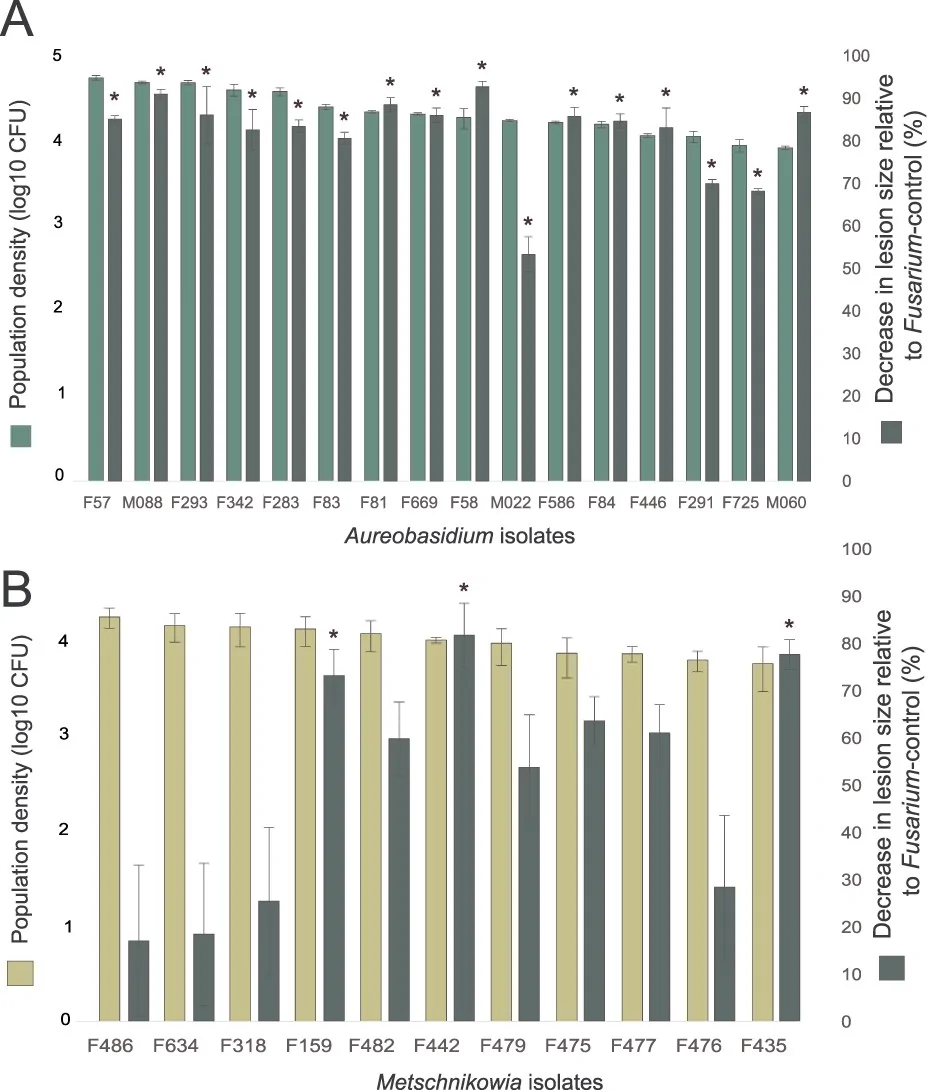
Intraspecific colonization and inhibition of *F. graminearum* by the wheat phyllosphere yeast isolates *Aureobasidium* and *Metschnikowia*. Yeast population densities during interaction with *F. graminearum* and growth inhibition of *F. graminearum* are shown for *Aureobasidium* (A) and *Metschnikowia* (B) isolates after 9 dpi. Green (*Aureobasidium* spp.) and yellow (*Metschnikowia* spp.) bars represent yeast leaf colonization (log10-transformed) in the presence of *F. graminearum* (log10 CFU/0.5cm^2^ lesion, mean ± SE). Grey bars represent inhibition percentage of *F. graminearum* (mean ± SE) relative to control (*Fusarium*-inoculated leaves). Each treatment consisted of three replicate plates from a detached leaf assay, each containing six leaves with two lesions per leaf (*n* = 3), except for F159 and F479 where one lesion, from one leaf, from one plate, was removed due to yellowing. Leaf lesion sizes were used as a proxy for pathogen growth and were calculated using Leica M205C and ImageJ. Statistical analysis was conducted using one-way ANOVA and Dunnett’s correction, and significant differences in lesion sizes between yeast-inoculated leaves and control (*F. graminearum* only) are indicated with an asterisk (*P* < .05).

We expanded the screening to all *Aureobasidium* and *Metschnikowia* isolates (Supplementary Table 1) in our culture collection using the detached leaf assay aiming to: (i) examine intraspecific variation in colonization persistence, (ii) determine the effect of *F. graminearum* on yeast population density, and (iii) evaluate the impact of yeasts on fungal invasion of *F. graminearum* and leaf infection. All isolates were able to colonize and proliferate on the leaves for a minimum of 7 days after inoculation with *F. graminearum*, reaching to a cell density of 2.8 × 10^4^ and 1.0 × 10^4^ CFU/0.5 cm^2^ lesion on average for *Aureobasidium* and *Metschnikowia* isolates, respectively (initial inoculum at *T*_0_ of ~100 CFU/0.5cm^2^ lesion prior to *F. graminearum* colonization) (Fig. 1A and B).

We also tested if inoculation with *F. graminearum* influenced the colonization of different yeast genera on wheat leaves, by comparing the cell densities on detached leaves in the presence and absence of *F. graminearum* at 9 dpi (7 dpi after fungal inoculation). Population densities of *Aureobasidium* isolates F446, M022, and M088 increased by 13%–33% after the inoculation of *F. graminearum*. A significant decrease was observed for isolate F81 (Welch *t-*test, *P* < .05; Supplementary Fig. 3A), while a significant decrease was observed for isolate F81 (Welch *t-*test, *P* < .05; Supplementary Fig. 3A). In addition, significant increases of up to 15-fold were observed for *Metschnikowia* isolates F318, F435, and F634 (>80%) (Student’s *t-*test for F318 and F634, and Welch two-sample *t-*test for F435; *P* < .05) (Supplementary Fig. 3B). We did not observe any detectable leaf colonization (detection limit: 100 CFU/0.5 cm^2^ lesion) by *Metschnikowia* isolate F435 in the absence of *F. graminearum* for the tested serial dilutions (10^3^–10^5^), whereas for all other isolates, we detected similar leaf colonization in the presence and absence of *F. graminearum* (Supplementary Fig. 3B). Disease control (i.e. reduced leaf lesion compared to *Fusarium*-inoculated control) differed between genera: all tested *Aureobasidium* isolates consistently and significantly reduced lesion size by >50%, whereas *Metschnikowia* isolates varied from 16%–80%, with only isolates F159, F442, and F435 significantly decreasing lesion size (Fig. 1A and B; one-way ANOVA, Dunnett’s correction, *P* < .05). No correlation between yeast population density and disease control (lesion size) was found for either *Aureobasidium* (*R*^2^*_Aureobasidium_* = 0.12, *P =* .18) or *Metschnikowia* (*R*^2^  *_Metschnikowia_* = 0.36, *P =* .05) (Supplementary Fig. 4A and B). For subsequent experiments, we selected *A. pullulans* F57 and *M. pulcherrima* F159 based on superior colonization, consistent disease suppression (>50%), and the availability of metabolic profiles [25].

**Figure 2:**
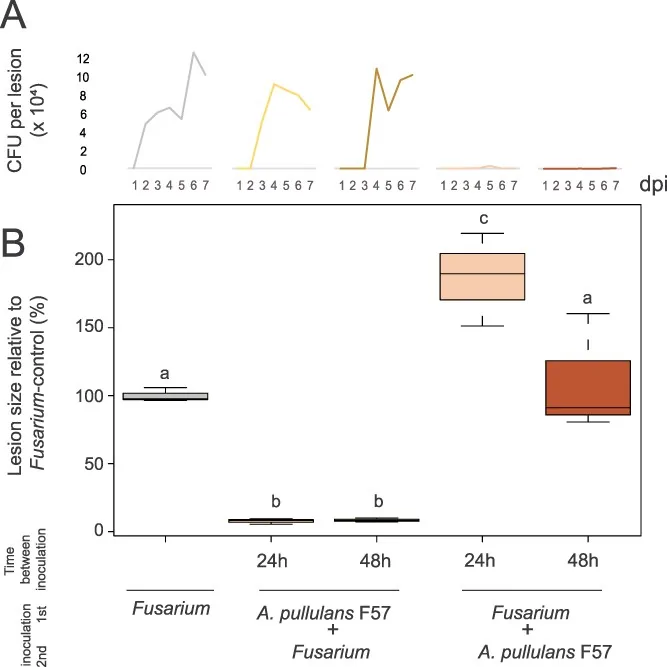
Priority effects during leaf colonization by *F. graminearum* and the yeast *A. pullulans* F57. Priority effects were tested using a detached leaf assay where both the fungal pathogen and the yeast were inoculated 24 and 48 h apart from each other, in both orders of arrival. (A) Colonization of *A. pullulans* F57 on wheat leaves before and after arrival of *F. graminearum.* The first panel (gray) represents *A. pullulans* F57 control treatment. Lesions were washed by vortexing and sonication and serial dilutions were prepared for a period of 7 days of (co-)inoculation, CFUs were determined for three lesions per treatment and timepoint. (B) Disease control was assessed photographing leaf samples with a Leica M205C stereomicroscope after 6 days and lesion size was measured by ImageJ software. Each replicate consisted of one plate containing 7 leaves with 2 lesions each (*n* = 3). Statistical differences (one-way ANOVA, and Tukey HSD, *P* < .05) are indicated by different letters on top of the bars (mean ± SE).

The timing of arrival of the yeasts and pathogen on the leaf surface, also referred to as the priority effect, strongly influences colonization. *Aureobasidium pullulans* F57 and *F. graminearum* were inoculated at 24 and 48 h apart in both orders of arrival. Colonization and disease incidence were assessed by dilution plating and lesion measurements (Fig. 2A and B; one-way ANOVA, Tukey HSD, *P* < .05). When *Aureobasidium* was inoculated before *F. graminearum*, only mild infections were observed, and yeast colonization matched levels seen without the pathogen. Conversely, when *F. graminearum* was inoculated first, leaf colonization by *Aureobasidium* was significantly reduced (i.e. lower cell densities), ranging from 1 × 10^2^–3 × 10^3^ CFU/0.5 cm^2^ lesion as compared to 5 × 10^4^–1 × 10^5^ CFU/0.5 cm^2^ lesion when inoculated 24–48 h prior to *F. graminearum*, respectively. Moreover, a significant increase in lesion size was observed (Fig. 2B; one-way ANOVA, Tukey HSD, *P =* .01).

### *Aureobasidium* and *Metschnikowia* colonize wheat heads and control FHB

To assess yeast colonization and competition with indigenous wheat head microbiota under field-like conditions, we conducted an outdoor pot experiment. Instead of dilution plating, we used RT-qPCR to discriminate between *Aureobasidium* and *Metschnikowia* yeasts, and other genera naturally occurring on heads of wheat plants grown outdoors. Genus-specific primers, designed to target *Aureobasidium* or *Metschnikowia*, showed no amplification in any of the nine nontarget yeast genera from the wheat phyllosphere. As RT-qPCR of RNA targets housekeeping genes of actively dividing cells, it served as a proxy for viable yeast density.

**Figure 3:**
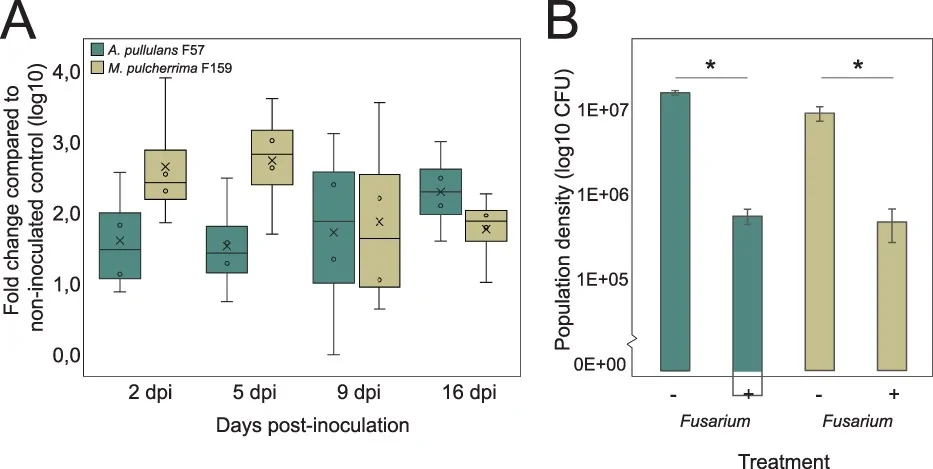
Colonization of phyllosphere yeasts on wheat heads in the presence and absence of *F. graminearum*. Wheat heads were spray-inoculated at the flowering stage with *A. pullulans* F57 and *M. pulcherrima* F159 in the presence and absence of *F. graminearum* in an outdoor pot experiment (plants were grown under natural conditions until flowering and then transferred to a covered tunnel for the inoculations) and a greenhouse pot experiment (plants were grown under controlled conditions). (A) Yeast colonization in an outdoor pot experiment under natural environment conditions for a period of 16 days, *n* = 4 for all treatments, except *A. pullulans* F57 at 9 dpi where *n* = 3. Dots in boxplots indicate individual data points; *x* indicates the mean value. Color legend applies to all panels. (B) Colonization of wheat heads by *A. pullulans* F57 and *M. pulcherrima* F159 in the presence and absence of *F. graminearum* in a greenhouse pot experiment. Population densities were determined after 14 days determined by plating (CFU counting, log10-transformed, mean ± SE). Sample sizes were *n* = 3 for all treatments. Statistical analysis based on Wilcoxon rank sum test, differences indicated by asterisk (*P* < .05).

Both isolates colonized the wheat heads in the detached wheat head assay and the outdoor pot experiment at 16 days post yeast-inoculation (Fig. 3A). For the detached head assay, we observed a 2900- and 80-fold higher population density in heads inoculated with *A. pullulans* F57 and *M. pulcherrima* F159 in the absence of *Fusarium*, based on RT-qPCR (Supplementary Fig. 5). In the outdoor pot experiment, the density of introduced *A. pullulans* F57 was 100–500-fold higher than the natural population (non-inoculated heads). The density of *M. pulcherrima* F159 was up to 2500-fold higher at 5 dpi compared to the natural population; however, colonization showed a gradual 100-fold decline at 16 dpi, possibly due to the high initial inoculation titer.

In addition to the outdoor pot experiment, a greenhouse experiment (greenhouse pot experiment 1) was performed to obtain absolute quantification in cell count, rather than relative quantification derived from RT-qPCR data. Sixteen days after inoculation of yeast isolates *A. pullulans* F57 and *M. pulcherrima* F159 under controlled greenhouse conditions in the absence of *F. graminearum*, CFU counts confirmed their ability to colonize and proliferate the wheat heads, reaching densities of 1.9 × 10^7^ and 1.1 × 10^7^ CFU/head, respectively (4- to 5-fold increase compared to the initial inoculum density), whereas only 3.5–7.5 × 10^4^ CFU/head were observed in the non-inoculated heads. In contrast, co-inoculation with *F. graminearum* reduced cell densities of *A. pullulans* F57 and *M. pulcherrima* F159 by 20- to 30-fold in greenhouse pot experiment 1, suggesting competition and/or antagonism between the yeasts and the fungal pathogen (Fig. 3B; Wilcoxon rank sum test; *P*_F57_ = .02, *P*_F159_ = .02).

**Figure 4:**
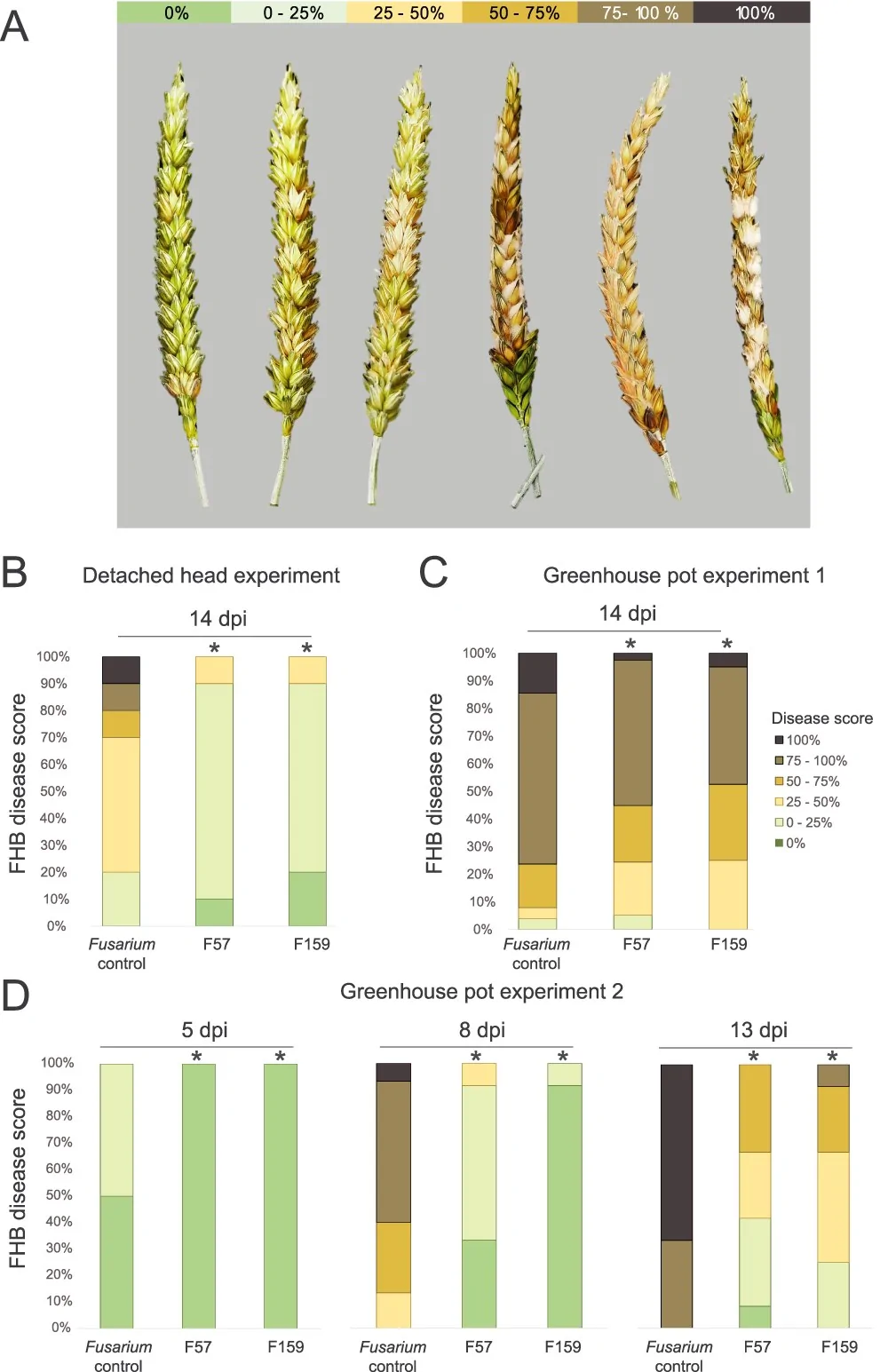
Incidence of Fusarium Head Blight (FHB) in wheat heads treated with phyllosphere yeasts. Disease of FHB incidence per wheat head was scored based on a six-level visual scale of increasing symptoms (e.g. bleaching and necrotic tissue) compared to control (i.e. wheat heads not treated with the yeasts and sprayed with *F. graminearum* only). (A) FHB disease scoring scale on wheat heads. Disease was scored based on the following disease scale: 0 = No visible symptoms; 1 = 0%–25% of bleached or necrotic areas; 2 = 25%–50%; 3 = 50–75%; 4 = 75%–100% and 5 = 100% bleached or necrotic areas and hyphae clearly growing outside of the heads. (B) FHB disease score at 14 dpi after fungal inoculation in a detached head experiment (*n* = 10). (C) FHB disease score at 14 dpi after fungal inoculation in greenhouse pot experiment (*n*_control_ = 76, *n*_Aureobasidium_F57_ = 78, *n*_Metschnikowia_F159_ = 40). (D) FHB disease score in a second greenhouse pot experiment monitored at 5, 8, and 13 days after fungal inoculation (*n* = 12, except for *n*_control_8dpi_ = 15). Statistical differences are indicated by an asterisk on top of the bars (Fisher exact test, Bonferroni, *P* < .05), only showing the statistical comparison between the *Fusarium*-inoculated heads (control) versus co-inoculated heads with *A. pullulans* F57 and *F. graminearum*, or *M. pulcherrima* and *F. graminearum*.

**Figure 5:**
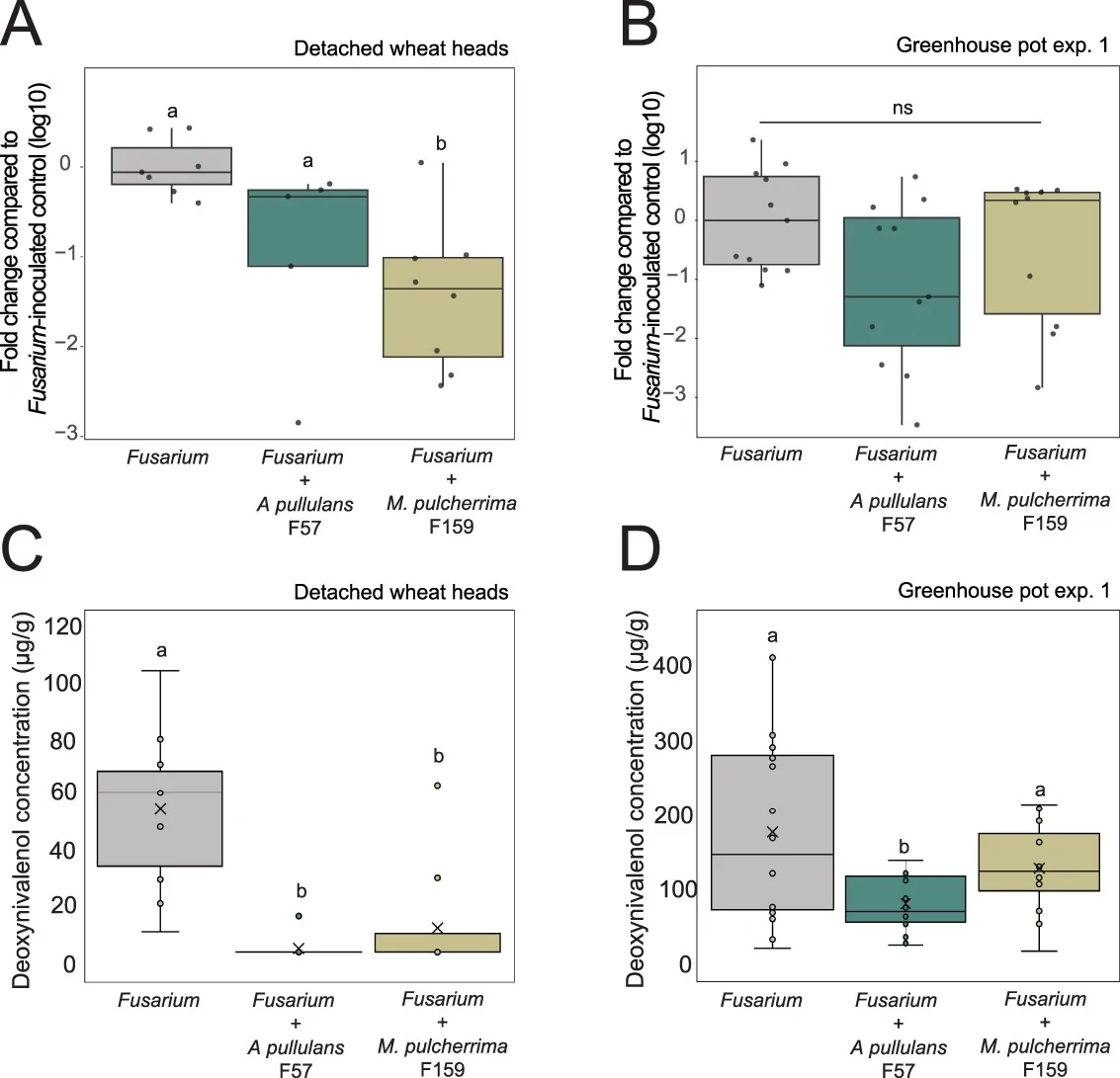
*Fusarium graminearum* biomass and deoxynivalenol (DON) concentrations detected on wheat heads treated with phyllosphere yeasts and *F. graminearum. Fusarium* biomass was quantified by RT-qPCR using the FaRa *Fusarium* housekeeping gene normalized to the act housekeeping gene present in wheat heads treated with *A. pullulans* F57 and *M. pulcherrima* F159 in: (A) the detached head assay (*n*_Fusarium_ = 7, *n*_F57_ = 5, *n*_F159_ = 8), where data were log10-transformed, and statistical differences were tested with one-way ANOVA, Dunnett’s correction (*P* < .05), and in (B) the greenhouse pot experiment 1 (*n*_Fusarium_ = 11, *n*_F57_ = 11, *n*_F159_ = 10), where data were log10-transformed, and statistical differences were tested with Kruskal–Wallis (*P =* .12). DON concentrations were determined by HPLC analysis of wheat heads pretreated with yeasts for 2 dpi from: (C) the detached head assay (mean ± SE, *n*_all_ = 10) (Kruskal–Wallis, Bonferroni, Dunn’s, *P* < .01) and (D) greenhouse pot experiment 1 (mean ± SE, *n*_control_ = 20, *n*_Aureobasidium_F57_ = 20, *n*_Metschnikowia_F159_ = 16). Statistical differences are indicated by different letters (one-way ANOVA, Tukey HSD, *P* < .01). Dots in boxplot indicate individual data points, *x* indicates the mean value.

We tested the impact of these yeast isolates on the invasiveness of *F. graminearum*, and subsequent wheat infection leading to FHB under greenhouse conditions using detached head and pot experiments. For the detached head experiment, flowering wheat heads were detached from whole plants. Clear FHB symptoms were observed in the *Fusarium*-inoculated control, including necrotic head tissue with outgrowing fungal hyphae at 14 dpi, whereas a significant reduction in FHB symptoms was observed for wheat heads inoculated with the yeast isolates (Fig. 4A and B; Fisher’s exact test, Bonferroni correction, *P* < .05). In the wheat heads treated with *A. pullulans* F57 and *M. pulcherrima* F159, a low disease severity was observed with the majority of the heads remaining healthy, with 90% of the heads showing 0% up to 25% infection. We observed a 2900- and 80-fold higher population density of *A. pullulans* 57 and *M. pulcherrima* F159 compared to non-inoculated heads, respectively, based on RT-qPCR (Supplementary Fig. 5). For the pot experiment (greenhouse pot experiment 1), mature wheat plants grown in soil were used. All yeast treatments significantly reduced FHB symptoms (Fig. 4C; Fisher’s exact test, Bonferroni correction, *P* < .01). An extra greenhouse experiment (greenhouse pot experiment 2) was conducted where disease symptoms were scored at different time points (5, 8, and 13 dpi). Significant reduction of FHB symptoms could be observed at all time points for both yeasts (Fig. 4D; Fisher’s exact test, Bonferroni correction; *P*_F57_5dpi_ = .03, *P*_F57_8dpi_ = 1.3 × 10^−6^, *P*_F57_13di_ = 5.9 × 10^−6^, *P*_F159_5dpi_ = .03, *P*_F159_8dpi_ = 4.6 × 10^−7^, *P*_F159_13 dpi_ = 5.3 × 10^−5^). No significant effect on alleviation of grain shivering caused by FHB disease was observed when *A. pullulans* F57 and *M. pulcherrima* F159 were inoculated (Supplementary Fig. 6; Mann–Whitney *U, P*_F57_ = 0.11, *Z*_F57_ = 1.586, *P*_F159_ = 0.49, *Z*_F159_ = −0.693). RT-qPCR analysis was used to quantify *Fusarium* biomass in both experiments. A reduction of 48% and a significant reduction of 63% was observed in detached wheat heads treated with *A. pullulans* F57 and *M. pulcherrima* F159, respectively (Fig. 5A; one-way ANOVA, Dunnett’s correction; *P*_F57_ = .09, *P*_F159_ = .005). A substantial but nonsignificant reduction of 53% and 29% was observed in mature wheat plants after *A. pullulans* F57 and *M. pulcherrima* F159 treatment, respectively (Fig. 5B; Kruskal–Wallis; *P =* .12). Additionally, expression of the cluster-specific transcription factor TRI6 in detached wheat heads was quantified by RT-qPCR as a marker of regulatory activation of the trichothecene mycotoxin biosynthetic gene cluster. Non-significant reductions of 34% and 14% were observed in *TRI6* gene expression after inoculation with *A. pullulans* F57 and *M. pulcherrima* F159, respectively (Supplementary Fig. 7; Kruskal–Wallis, *P* > .05).

### *Aureobasidium* and *Metschnikowia* reduce deoxynivalenol levels

To evaluate the effect of yeast inoculation on deoxynivalenol (DON) concentration on wheat head infected with *F. graminearum*, we measured the DON concentration using detached head and greenhouse pot experiment 1. Detached wheat heads infected with *F. graminearum* accumulated an average DON concentration of 53.9 (±9.3) μg/g at 14 dpi (Fig. 5C). When wheat heads were pretreated with either *A. pullulans* F57 and *M. pulcherrima* F159, DON concentrations were significantly reduced to 2.3 (±1.3) and 9.9 (±6.5) μg/g (Kruskal–Wallis, Bonferroni, Dunn’s, *P*_F57_ = 1.5 × 10^−6^, *P*_159_ = 5.9 × 10^−5^), corresponding to 96% and 82% decrease compared to the non-treated heads, respectively. Reductions in DON concentrations were also observed in the whole plant greenhouse pot experiment 1, where *Fusarium*-infected wheat heads accumulated an average DON concentration of 175.8 (± 28.1) μg/g head tissue, with heads pretreated with *A. pullulans* F57 contained 55% [79.1 μg/g (± 8.1)]. Pretreatment with *M. pulcherrima* F159 did not significantly influence DON accumulation [−26% DON; 126.8 μg/g (± 14.8)] (Fig. 5D; one-way ANOVA, Tukey HSD; *P*_F57_ = .001, *P*_F159_ = .20).

**Figure 6:**
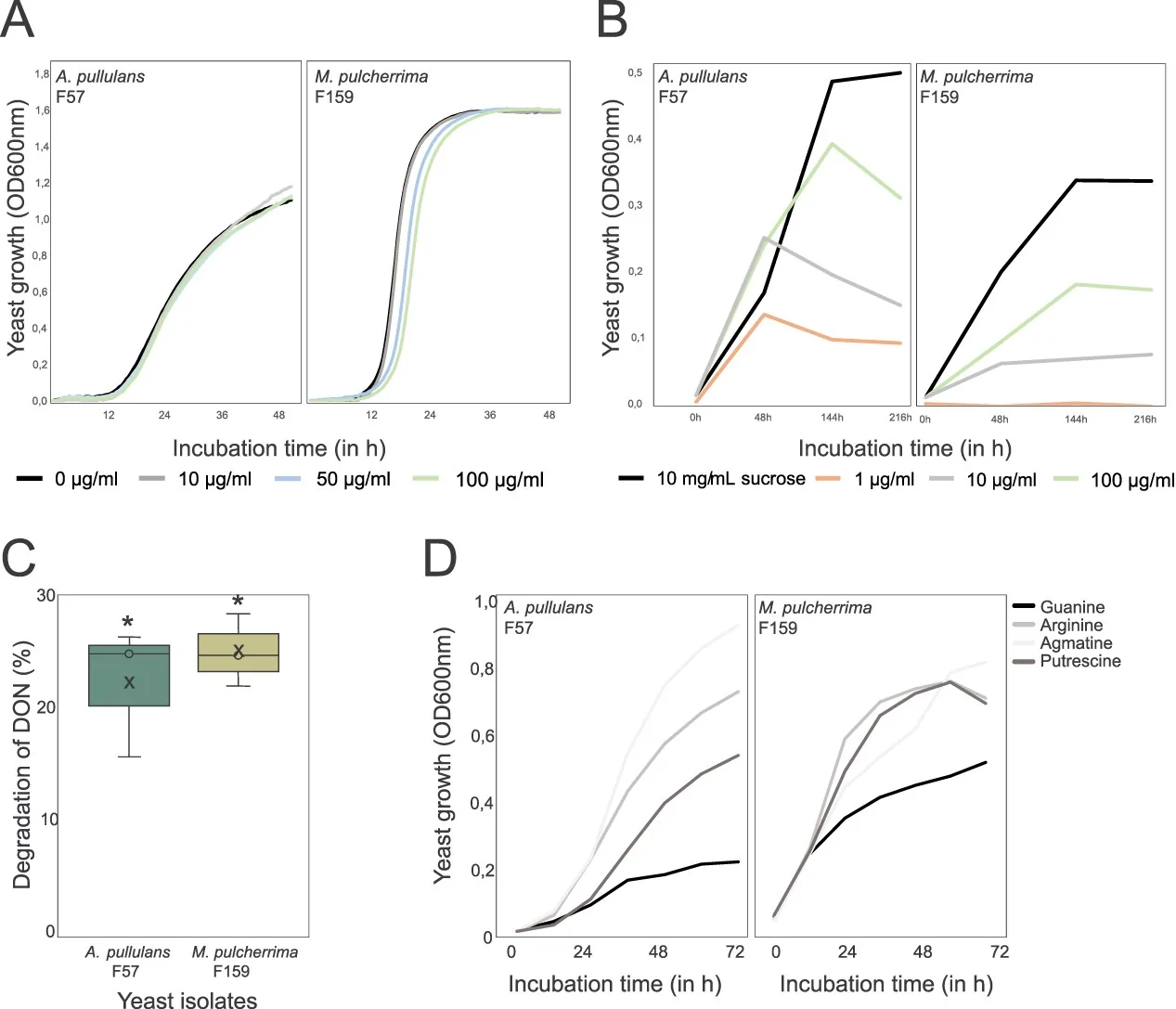
Yeast growth on deoxynivalenol (DON) and on plant-derived amino acids and amine during *F. graminearum* infection of wheat heads. A) in vitro growth of *A. pullulans* F57 and *M. pulcherrima* F159 in Potato Dextrose Broth (PDB) medium supplemented with ranging DON concentrations (0, 10, 50, and 100 μg/ml). Measurements were taken every 30 min at OD_600nm_ (*n* = 3). (B) *In vitro* growth of *A. pullulans* F57 and *M. pulcherrima* F159 in minimal medium supplemented with ranging DON concentrations (1, 10, and 100 μg/ml) or sucrose (μg/ml, 1%), measurements were taken at OD_600nm_ after 0, 48, 144, and 216 h (*n* = 3). (C) Quantification of DON degradation (mean ± SE, *n* = 3) by HPLC-UV analysis after 216 h in minimal media supplemented with DON (100 μg/ml). Statistical difference in recovery percentage of DON on yeast-inoculated samples compared to control (noninoculated sample supplemented with 100 μg/ml DON) is indicated with an asterisk (one tailed *t-*test, *P* < .05). (D) *In vitro* growth of *A. pullulans* F57 and *M. pulcherrima* F159 in minimal medium supplemented with different amino acids or one polyamine at 5 mM. Measurements were taken every 12 h at OD_600nm_ (*n* = 3).

To explore how yeasts reduce mycotoxin concentrations, we tested whether *A. pullulans* F57 and *M. pulcherrima* F159 tolerate or degrade DON. Both isolates were grown in minimal and rich medium (PDB) with DON concentrations ranging from 1 to 100 μg/ml. *Aureobasidium pullulans* F57 showed no growth inhibition at any of the tested DON concentrations on the rich media (Fig. 6A), whereas *M. pulcherrima* F159 showed a growth delay of ~2 and 3 h was observed upon the addition of 50 and 100 μg/ml DON, respectively. However, the growth rate returned to levels observed in unamended media after 3 h. When grown on the minimal media where DON is used as the sole carbon source, *A. pullulans* F57 showed a higher growth rate with increasing DON concentrations compared to the lowest concentration (1 μg/ml) of DON and sucrose (1%) (Fig. 6B). When supplemented with 1 or 10 μg/ml of DON, *A. pullulans* F57 maintained growth for 48 h, whereas at 100 μg/ml of DON, growth extended to 144 h before declining. In contrast, *Metschnikowia* showed no growth at low DON concentration (1 μg/ml), but increasing growth rates at higher concentrations (10 and 100 μg/ml), albeit at slower growth rates than in sucrose.

To test if yeasts were able to degrade DON *in vitro*, samples for HPLC-UV analysis were collected in minimal medium with 100 μg/ml DON as the sole carbon source for 216 h (9 days), corresponding to an OD_600nm_ of ~0.3 for both yeast isolates after 9 days (Fig. 6B). We found a significant degradation rate of 21.9% (± 2.8) and 26.1% (± 1.5) for *A. pullulans* F57 and *M. pulcherrima* F159 on average, respectively (Fig. 6C; *t-*test; *P*_F57_ = .04, *t*-value_F57_ = 2.45 and df_F57_ = 5, *P*_F159_ = .03, *t*-value_F159_ = 2.64 and df_F159_ = 5).

To explore a potential mitigation strategy to alleviate disease severity, we investigated whether the yeast isolates could utilize plant defense-related compounds, linked to the induction of TRI gene expression and DON biosynthesis in *F. graminearum*, as sole nitrogen source (at 5 mM). These compounds were the nitrogen-rich plant-derived amino acids such as guanine (C_5_H_5_N_5_O), arginine (C_6_H_14_N_4_O_2_), the amino acid derivative agmatine (C_5_H_14_N_4_), and the polyamine putrescine (C_4_H_12_N_2_) [33]. *Aureobasidium pullulans* F57 and *M. pulcherrima* F159 were able to grow on all four plant derived compounds (Fig. 6D). We observed different metabolic ability of the yeast isolates to utilize the nitrogen sources, with fastest growth rate found on agmatine, followed by arginine, putrescine, and guanine, despite guanine providing the highest number of available nitrogen atoms. *Aureobasidium pullulans* F57 reached OD_600nm_ between 0.23 and 0.95 and *M. pulcherrima* F159 reached OD_600nm_ between 0.54 and 0.84 after 72 h of incubation. Collectively, these results indicate that these two yeast isolates can utilize plant-derived metabolites that trigger DON production and can use DON as a sole carbon source.

### Yeasts mitigate plant metabolic stress responses to *Fusarium* infection

**Figure 7:**
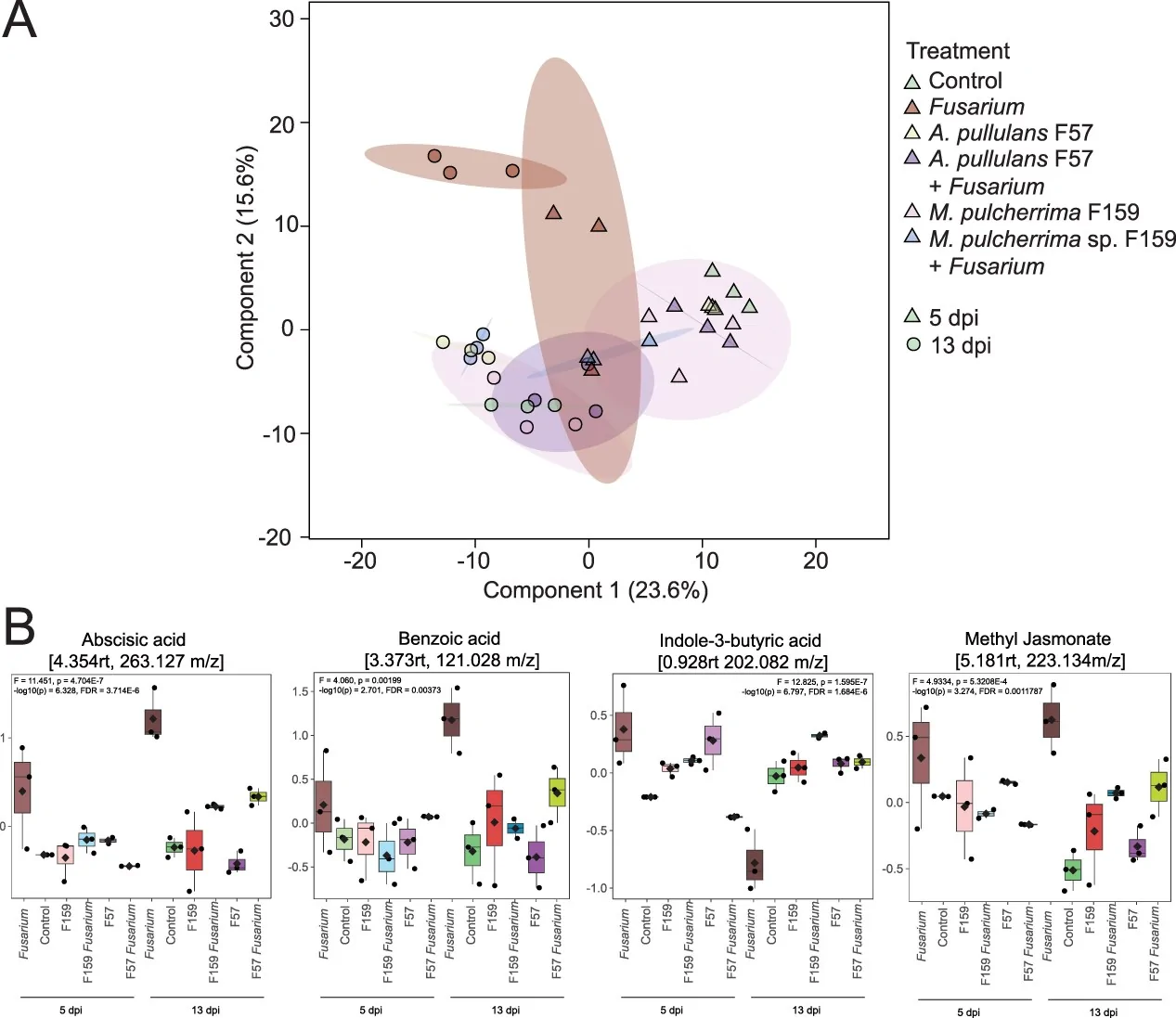
Modulation of plant hormones and phenolic compounds by *fusarium graminearum* and phyllosphere yeasts. PLS-DA analysis illustrating separation of treatments based on metabolome of (A) *M. pulcherrima* F159– and *A. pullulans* F57–inoculated heads in the presence and absence of *F. graminearum*. Control represents noninoculated wheat heads. Samples were taken after 5 and 13 days post-*Fusarium* inoculation. Each treatment represents three independent replicates (plants). Triangles (5 dpi) and circles (13 dpi) represent days post-*Fusarium* inoculation, color-coded based on inoculum and applies to all panels. Ovals represent 95% confidence ellipses around each group its centroid. (B) Plant hormones and phenolic compounds detected in untargeted LC-MS/MS analysis (mean ± SE). All data were log-transformed and Pareto-scaled prior to visualization and performed using Metaboanalyst v6.0 (one-way ANOVA, Tukey HSD, *P* < .05).

We aimed to investigate the influence of yeast or *Fusarium* inoculation on the wheat metabolome. For that, untargeted metabolomic profiling of wheat heads (greenhouse pot experiment 2) was performed to analyze the effect of yeast-, pathogen-, and their co-inoculation. A total of 1595 compounds were detected, of which 158 were annotated based on MS/MS spectra (Supplementary Table 2). First, we investigated the dipartite interactions between the host (non-inoculated wheat control) and yeast as well as the host and *Fusarium. Fusarium* infection accounted for most of the observed metabolomic alteration, whereas yeast-inoculation alone, especially inoculation with *M. pulcherrima* F159, had little effect on the wheat metabolome (*t-*test, *P*_Fusarium_ < .05; 281 significantly different compounds, *P*_F159_ < .05; 10 compounds; *P*_F57_ < .05; 80 compounds) (Fig. 7A, Supplementary Table 3). Only a limited number of significantly different metabolites (*t-*test, *P* < .05; 40 compounds) were identified during co-inoculation of *Fusarium* with *A. pullulans* F57, or *Fusarium* with *M. pulcherrima* F159, of which only two could be annotated (4-hydroxybenzoic acid and guanosine) (Supplementary Table 3), highlighting the similar metabolic impact of these yeasts on plant metabolic profiles. Consequently, we focused on the analysis of metabolites associated with plant health as a first step into unraveling the molecular mechanisms underlying plant protection against FHB by yeasts.

Targeted profiling revealed distinct changes in key phytohormones and phenolic compounds in wheat heads upon *Fusarium* infection (Fig. 7B). We observed significant increases in abscisic acid (ABA), benzoic acid, and methyl jasmonate levels, and a decrease in indole-3-butyric acid (IBA). After 13 dpi, *Fusarium*-inoculated heads contained significantly higher concentrations of ABA (*P =* .01, *Z* = 24.47), methyl jasmonate (*P =* .001, *Z* = 31.4), and benzoic acid (*P =* .01, *Z* = 25.2), compared to noninoculated heads. In wheat heads co-inoculated with *Aureobasidium*–*Fusarium* or *Metschnikowia*–*Fusarium*, levels of ABA (*P*_F57_ = .09, *Z*_F57_ = 16.8, *P*_F159_ = .25, *Z*_F159_ = 11.5), benzoic acid (*P*_F57_ = .14, *Z*_F57_ = 1.45; *P*_F159_ = .65, *Z*_F159_ = 0.46), methyl jasmonate (*P*_F57_ = .04, *Z*_F57_ = 20.6; *P*_F159_ = .06, *Z*_F159_ = 19.1), and IBA (*P*_F57_ = .40, *Z*_F57_ = 0.84; *P*_F159_ = .02, *Z*_F159_ = 22.9) were similar as those observed in non-inoculated control heads, except for methyl jasmonate in *Aureobasidium-Fusarium* co-inoculated heads, and IBA in *Metschnikowia-Fusarium* co-inoculated heads (Fig. 7B, Kruskal–Wallis, Bonferroni, Dunn’s; *P* < .05). This suggests mitigation of stress and defense responses typically triggered by *Fusarium* infection, upon yeast colonization. In addition, this regulatory effect highlights the potential of phyllosphere yeasts to influence host–pathogen interactions beyond direct antagonism, possibly through plant hormonal crosstalk or priming mechanisms.

## Discussion

In this study, we investigated the phyllosphere competence of different yeasts and their competitive interactions with the mycotoxigenic fungus *F. graminearum*. The results showed that *Aureobasidium* and *Metschnikowia* isolates, originally isolated from wheat flag leaves, can successfully colonize and proliferate on wheat leaves and heads and reduce FHB symptoms and mycotoxin levels. Different colonization dynamics were observed for these yeasts, with higher population densities for *Metschnikowia* isolates during co-inoculation with *F. graminearum* on wheat leaves compared to single inoculation (i.e. no *F. graminearum*). This higher colonization is most likely due to the leakage of nutrients from cells infected by the fungal pathogen [38]. Although *Aureobasidium* isolates showed higher cell densities than *Metschnikowia* on leaves, their colonization dynamics was not affected by the fungal pathogen. The more prolific growth of *Aureobasidium* isolates in the leaf environment may be due to their more flexible carbon utilization spectrum as compared to *Metschnikowia* [25]*.* In this context, also priority effects will play an important role in community assembly and pathogen antagonism. Our results showed that the order of arrival was critical for the interactions between *A. pullulans* F57 and *F. graminearum*. Significant pathogen inhibition was observed only when the yeast isolate was inoculated at least 24 h prior to *F. graminearum*. This early arrival, also referred to as niche pre-emption, allows species to occupy space and utilizing resources before others establish [23]. Additionally, early-arriving species may already be producing secondary metabolites, thereby altering the chemical landscape on the leaf and impacting the growth of the late-arriving species. Conversely, a significant increase in lesion size was observed when *F. graminearum* was inoculated 24 h prior to *Aureobasidium*. We hypothesize that *Aureobasidium* influences other resident microorganisms, thereby altering the microbial community structure in a way that creates additional ecological space for *F. graminearum* to proliferate. Although the underlying mechanism remains unclear, this observation may reflect stress-induced increase in pathogen aggressiveness when the antagonist is encountered after infection establishment. Similar responses have been described following exposure of *Fusarium* to sublethal levels of azoles, which can induce TRI gene expression and increase trichothecene production, thereby enhancing virulence and disease severity [39]. Metabolomic identification of the nutrients present on healthy leaves and leaking from leaves upon *Fusarium* infection will be crucial to further investigate the molecular basis of the observed priority effects in this yeast–pathogen interaction. Also, amendment of specific substrates could be conducted to investigate whether a surplus of these nutrients would affect the outcome of the yeast–pathogen interactions. Another important step will be to investigate whether this phenomenon persists under field conditions, where the initial total microbial biomass is likely to be significantly higher and the yeast establishment might face competitive pressure.

Earlier studies on *A. pullulans* hypothesized that it competes with pathogens for space and nutrients [16]. However, recent studies based on multi-omics suggested additional mechanisms involving the induction of the plant immune response and enhanced levels of the plant defense–hormone salicylic acid [22]. The plant genes *PR1* and *PR2*, involved in hormone-dependent plant immune activation, showed a 5- to 10-fold increase after application of *A. pullulans*. For *Metschnikowia* isolates, previous studies have shown that they compete for glucose and fructose to suppress the fungal pathogen *Colletotrichum gloeosporioides* in mango fruits [40, 41]. More specifically, high-capacity amino acid transporter genes (*GAP1* and *PUT4*) were proposed to aid in the priority effect of *M. reukaufii* against other nectar microorganisms, such as *Candida rancensis* [24]. Although this observation was made in nectar-associated microbial communities rather than in plant–pathogen interactions, it suggests that efficient nutrient scavenging may represent a more general competitive strategy that could also influence yeast establishment and resource competition during interactions with *F. graminearum*. Preliminary genome mining of the yeast isolates used in our study identified the presence of these two transporter genes in *M. pulcherrima* F159 (Supplementary Table 4). Transcriptome analyses and mutagenesis of specific yeast genes expressed during colonization and interaction with *F. graminearum* will be required to further unravel their involvement in the mode(s) of action in yeast–*Fusarium* interactions.

*Fusarium graminearum* and phyllosphere yeasts display different niche occupancy [2, 42]. *Fusarium graminearum* initially colonizes the surface of wheat heads, subsequently developing runner hyphae that penetrate the inner tissue of the spikelets and establish endophytic growth. In contrast, yeasts typically colonize the phyllosphere epiphytically. We observed a 2- and 5-fold increase in CFU after 14 days (compared to the initial inoculum density) for *A. pullulans* F57 and *M. pulcherrima* F159, respectively, demonstrating their ability to also colonize and proliferate on the wheat heads [25]. Upon confrontation with *F. graminearum*, we observed a 10-fold decrease in colonization of the wheat heads for both yeast isolates, whereas confrontation on detached leaves did not show a significant reduction in yeast colonization. The wheat head environment, the main infection court of *F. graminearum*, may be more conducive to *F. graminearum* growth than the leaf surface or may produce different metabolites in the wheat heads than on the leaf that, in turn, may adversely affect growth of the yeasts. Additionally, in the detached leaf assay, yeast–pathogen interaction is forced within the confined inoculation site, whereas spray inoculation of the wheat heads allows for greater niche separation [43, 44]. *Fusarium graminearum* may also impact the colonization of *Aureobasidium* and *Metschnikowia* indirectly by altering the microenvironment on the wheat heads, for example, by affecting the pH via secretion of organic acids [45].

The main concern regarding FHB is the accumulation of mycotoxins, such as DON [46]. DON has important ecological roles, including a role in virulence and in competitiveness. For example, DON has been shown to be toxic to eukaryotes and to repress chitinase and glucanase gene expression in *Trichoderma atroviride* [47, 48]. Here we showed that both yeast isolates reduced DON levels in (detached) wheat heads by up to 95%, as well as a 48%–63% decrease in *Fusarium*-biomass after yeast introduction in detached grains and a 29%–53% decrease in whole plants. In contrast, *TRI6* gene expression exhibited only a minor nonsignificant downregulation. The greater reduction in DON levels compared to *Fusarium* biomass may be attributed to differences in measurement approaches: *Fusarium* biomass was assessed based on actively transcribing cells (RNA), whereas DON quantification reflects the cumulative mycotoxin accumulation over time. Because *TRI6* gene expression is highly dynamic and strongly dependent on the specific sampling timepoints, its measurement may underestimate the overall impact of yeast treatment. By contrast, DON levels integrate both the extent and duration of fungal toxin production, making them a more robust and biologically meaningful indicator. Additionally, both yeasts were able to grow in vitro with DON as the sole carbon source. Although DON did not impact the growth of *A. pullulans* F57, it slightly affected growth of *M. pulcherrima* F159. How both yeast isolates metabolize DON is not yet known. Previous studies have shown that DON can also be absorbed by the cell wall or be transformed into less toxic derivatives as part of detoxification mechanisms (e.g. acetylation, hydrolyzation, and glycosylation) [49, 50]. *Aureobasidium* and *Metschnikowia* isolates were both able to degrade DON, though to a limited extent, likely due to restricted yeast growth. Therefore, we hypothesized that DON degradation could be more significant under conditions providing additional nutrients, leading to increased biomass. This hypothesis is supported by previous work, which demonstrated the crucial role of media composition in DON detoxification by bacteria. Among the nine media tested, bacterial isolates exhibited de-epoxylating capabilities exclusively in Nutrient Broth and Mineral Salts Broth [51].

DON degradation most likely involves a broad range of genes, including those related to metabolism and transporters [52]. More specifically, disruption and overexpression studies have highlighted the importance of ABC transporters and major facilitator superfamilies (MFSs) in sensitivity to DON. Genes such as *Sts1, TRI12,* and *PDR* play a crucial role in multi-drug sensitivity of the model yeast *S. cerevisiae,* including DON sensitivity [53–56]. *TRI12* encodes a trichothecene efflux pomp, whereas *PDR5* encodes an ABC superfamily transporter known for mediating trichothecene resistance. Furthermore, the overexpression of *Sts1* was suggested to have a potential role in the innate defense or detoxification processes within growing yeast cells. Preliminary genome mining of *A. pullulans* F57 and *M. pulcherrima* F159 revealed the presence of a *Sts1/Cut8* complex as well as *TRI12* and PDR-like domains (Supplementary Table 4). More specifically, *Aureobasidium* sp. F57 encoded 18 genes annotated as *TRI12* and only one of those genes could be identified in *M. pulcherrima* F159. This difference might explain the insensitivity of *A. pullulans* F57 to high DON concentrations. However, whether these genes are involved in DON degradation by the yeast isolates requires further validation through genome-wide random mutagenesis, transcriptomic, and gene knockout approaches.

Specific host plant signals—especially those involved in the stress response—can promote DON production by the pathogen, including phytochemicals, H_2_O_2,_ and specific amino acids. The addition of amines, such as putrescine, and amino acids as nitrogen sources, strongly induce the *TRI5* gene, which is involved in the biosynthesis of DON [34]. Given that DON serves as a virulence factor for *F. graminearum*, increased DON production triggers a higher plant stress response that may lead to enhanced availability of free putrescine and consequently more DON. The observed difference between detached head and whole plant assays could therefore reflect differences in plant metabolic status or systemic signaling. Although detached heads may still accumulate putrescine locally in response to infection, they lack the full systemic context of an intact plant, which could influence both polyamine metabolism and fungal behavior. However, this remains speculative, and direct measurements of putrescine levels in both systems would be needed to confirm this hypothesis. Our results from the growth assays in minimal medium supplemented with four of host-derived compounds known to trigger DON biosynthesis in *F. graminearum* (guanine, arginine, agmatine, and putrescine) highlighted the ability of both *A. pullulans* F57 and *M. pulcherrima* F159 to use these compounds as sole nitrogen sources, potentially aiding the plant in its protection against FHB infection.

Shifts in plant metabolomic profiles are consistent with known plant responses to biotic stress: accumulation of ABA and jasmonates is often associated with stress signaling and immune modulation, and benzoic acid as a precursor to salicylic acid and antimicrobial metabolites [57, 58]. The role of the auxin-related compound indole-3-butyric acid is less well studied. IBA functions as a precursor or storage form of indole-3-acetic acid (IAA) and contributes to root development; however, its specific role during plant-pathogen interactions is not well understood [59, 60]. In general, the role of auxin signaling during pathogen infection is context-dependent, varying with both the type of pathogen and the nature of the interaction, and could either contribute to defense responses or promote susceptibility [59]. IBA biosynthesis in plants such as maize involves conversion from IAA, linking these hormones tightly in auxin homeostasis and signal pathways [59]. Our untargeted metabolomic analyses demonstrated the impact of FHB infection on the wheat metabolome. A reduction in IBA in *Fusarium*-infected heads could be observed at 13 dpi, which could reflect disruption of growth and developmental processes caused by pathogen-induced stress or tissue damage. Whether the decrease in IBA is a consequence of fungal colonization, a trigger of defense signaling, or a byproduct of declining tissue viability [60] remains unknown. Co-inoculation with yeasts mitigated *Fusarium*-induced metabolomic changes, restoring metabolite profiles to levels comparable to those observed in the noninoculated (control) heads. This indicates a buffering effect by yeasts on *Fusarium*-triggered metabolic stress or a reduction of pathogen pressure to levels below the threshold needed to activate robust plant defenses.

## Concluding remarks

This study provides a comprehensive exploration of the interaction dynamics between indigenous wheat phyllosphere yeasts and the fungal pathogen *F. graminearum*, with implications for new sustainable strategies in agriculture. We demonstrate that the newly isolated *Aureobasidium* and *Metschnikowia* isolates can effectively colonize wheat leaves and heads, diminish FHB symptoms and mycotoxin (DON) accumulation. The observed strain-specific variations in disease suppression and the role of priority effects underscore the complexity of microbial interactions within the phyllosphere. These findings highlight the ecological significance of niche pre-emption and resource competition in shaping microbial community structure and function.

## Supplementary Material

Supplementary_material_wrag187

## Data Availability

All data necessary to support the conclusions are included in the main text and/or the Supplementary Materials.
