# Supplementary material for: Yeast–Fusarium interactions and mycotoxin modulation in the phyllosphere
Source: ISME J. 2026 Jul 16;20(1):wrag187. doi: 10.1093/ismejo/wrag187 (PMC13528865; doi:10.1093/ismejo/wrag187)
Supplement: Supplementary file 1 [file supplementary_material_wrag187.zip › Supplementary Table 1_revised.pdf]

Supplementary Table 1. Yeast isolates used in this study, indicating isolate ID, genera, species, and source. Species identification was based on ITS rRNA gene sequencing.

Isolate ID	Genera	Species (based on ITS)	Source	Only co-inoculation w/ *F.*	Single & co-inoculation	Priority effect	CFU counting	RT-qPCR primer design
F57	*Aureobasidium*	*pullulans*	Gouka et al., 2022		x	x	x	
F174	*Aureobasidium*	*pullulans*	Gouka et al., 2022	x				x
F359	*Aureobasidium*	*pullulans*	Gouka et al., 2022	x				
F159	*Metschnikowia*	*pulcherrima*	Gouka et al., 2022		x		x	
F318	*Metschnikowia*	*pulcherrima*	Gouka et al., 2022		x		x	
F327	*Papiliotrema*	*flavescens*	Gouka et al., 2022	x				
F347	*Papiliotrema*	*flavescens*	Gouka et al., 2022	x				
F297	*Papiliotrema*	*flavescens*	Gouka et al., 2022					x
F179	*Sporobolomyces*	*roseus*	Gouka et al., 2022	x				
F185	*Sporobolomyces*	*roseus*	Gouka et al., 2022					x
F314	*Vishniacozyma*	*tephrensis*	Gouka et al., 2022					x
F345	*Vishniacozyma*	*tephrensis*	Gouka et al., 2022	x				
F294	*Holtermanniella*	*festucosa*	Gouka et al., 2022					x
F391	*Cystobasidium*	*slooffiae*	Gouka et al., 2022					x
F311	*Filobasidium*	*globisporum*	Gouka et al., 2022					x
F136	*Pseudotremella*	*moriformis*	Gouka et al., 2022					x
F224	*Rhodotorula*	*glutinis*	Gouka et al., 2022					x
F58	*Aureobasidium*	*pullulans*	This study		x		x	
F81	*Aureobasidium*	*pullulans*	This study		x		x	
F83	*Aureobasidium*	*pullulans*	This study		x		x	
F84	*Aureobasidium*	*pullulans*	This study		x		x	
F283	*Aureobasidium*	*pullulans*	This study		x		x	
F291	*Aureobasidium*	*pullulans*	This study		x		x	
F293	*Aureobasidium*	*pullulans*	This study		x		x	
F342	*Aureobasidium*	*pullulans*	This study		x		x	
F446	*Aureobasidium*	*pullulans*	This study		x		x	
F586	*Aureobasidium*	*pullulans*	This study		x		x	
F669	*Aureobasidium*	*pullulans*	This study		x		x	
F725	*Aureobasidium*	*pullulans*	This study		x		x	
M022	*Aureobasidium*	*pullulans*	This study		x		x	
M060	*Aureobasidium*	*pullulans*	This study		x		x	
M088	*Aureobasidium*	*pullulans*	This study		x		x	
F435	*Metschnikowia*	*viticola*	This study		x		x	
F442	*Metschnikowia*	*viticola*	This study		x		x	
F475	*Metschnikowia*	*viticola*	This study		x		x	
F476	*Metschnikowia*	*viticola*	This study		x		x	
F477	*Metschnikowia*	*viticola*	This study		x		x	
F479	*Metschnikowia*	*viticola*	This study		x		x	
F482	*Metschnikowia*	*viticola*	This study		x		x	
F486	*Metschnikowia*	*viticola*	This study		x		x	
F634	*Metschnikowia*	*viticola*	This study		x		x	
