# Supplementary material for: Yeast–Fusarium interactions and mycotoxin modulation in the phyllosphere
Source: ISME J. 2026 Jul 16;20(1):wrag187. doi: 10.1093/ismejo/wrag187 (PMC13528865; doi:10.1093/ismejo/wrag187)
Supplement: Supplementary file 1 [file supplementary_material_wrag187.zip › 31.03.2026_Revised Supplementary Material.docx]

**Metabolome analysis of wheat heads**

Wheat heads from greenhouse pot experiment 2 were ground into a fine, homogeneous powder with a Qiagen TissueLyser II bead mill at 30 Hz for 1.5 min. Fifty mg of the homogeneous plant material was weighed into a 2 mL Eppendorf tube containing 1 mL of pre-cooled 80% methanol (LC-MS grade, Chromasolv®). The tubes, placed in pre-cooled racks at -20 °C, were fitted with two glass beads and extracted for 3 min at an oscillation frequency of 30 Hz. The samples were then centrifuged at 20,000 g for 10 min at 4°C.

Secondary metabolites extracted from wheat heads were analysed using a binary Acquity UHPLC I-Class system hyphenated to a Synapt G2-S HRMS QTOF mass spectrometer (Milford, MA, USA). Separation was performed on an ACQUITY UPLC BEH C18 column (2.1 mm × 100 mm, 130Å, 1.7 µm) with a VanGuard pre-column (BEH C18, Waters), thermostated at 40 °C with a flow rate of 0.3 mL/min. The gradient program was as follows: initial conditions of 95% eluent A (MilliQ H2O + 0.1% FA) held for 30 s, followed by a linear increase to 30% eluent B (methanol + 0.1% FA) over 4 minutes, and a further increase to 95% eluent B over 2.5 min. This condition was maintained for 2.5 min, then returned to the starting conditions with a 2-minute equilibration, resulting in a total run time of 10 minutes per sample. The autosampler was kept at 4°C with an injection volume of 1.5 µL for all samples.

Detection was carried out with electrospray ionization (ESI) on a Synapt G2-S quadrupole time-of-flight mass spectrometer (QTOFMS) (Waters) in both positive (ESI+) and negative (ESI–) modes over a mass range of 50 to 1200 m/z. For ESI+, the parameters were as follows: capillary voltage of 2500 V, source temperature of 120°C, desolvation temperature of 500°C, cone gas flow of 50 L/h, desolvation gas flow of 1000 L/h, and nebulizer gas pressure of 5.0 bar. Samples were run in Data-Independent-Acquisition (DIA) (MS*^E^* on the Waters QTOFMS system) centroid mode with a scan time of 0.35 seconds and ramped trap collision energies from 10 to 50 V. Leucine enkephalin was used for internal lock mass calibration with m/z 554.2615 for [M-H]⁻ in ESI-, with mass calibration performed every 30 seconds. For quality control, QC samples were prepared by mixing 50 µl of each extract and used for correcting instrumental signal drift over the time. Samples were randomly distributed across batch. After data acquisition, manual inspections of base peak ion (BPI) chromatograms were performed to evaluate data quality based on QC runs, including assessments of retention time shifts, m/z deviations, and intensity thresholds for both MS1 and MS2.

The vendor–specific data files (Waters .raw) were exported to an open source software AnalysisBaseFileConverter (<https://www.reifycs.com/AbfConverter/>) for conversion of LC-MS raw data files to the open data format .abf which enables the data analysis in vendor-independent environment. The converted files were subjected to MS-DIAL version 4.92 (<https://prime.psc.riken.jp/compms/msdial/main.html>) pipeline, which allows the work with DIA data, since implemented MS^2^Dec algorithm for peak deconvolution can handle difficulties related to DIA data. Peak detection, including smoothing, was conduct according to the MS-DIAL algorithm, which consists of differential calculus and noise estimations^36^. In next step, the ‘peak spotting’, based on RT, and MS1 data axes were performed resulting the formation of BPI chromatograms for each individual mass slice of 0.1 m/z with a step size of 0.05 m/z for both datasets. Subsequently, MS2Dec deconvolution was applied to all detected ‘spots,’ followed by peak alignment and interpolation of missing values. Afterwards the drift correction within a batch was applied on the imputed data matrix of detected and aligned peaks to remove the effects of peak intensity drifts throughout the analytical sequence. Drift correction was performed with LOWESS (locally weighted scatterplot smoothing) algorithm to normalize the drifts present in MS signal measurements.

The tables of drift-corrected features were imported to MS-CleanR (<https://github.com/eMetaboHUB/MS-CleanR>) R-based feature-filtering workflow for untargeted LC–MS Based Metabolomics based on MS-DIAL pipeline^37^. The parameterization for data treatment of secondary metabolites were as follow: minimum blank ratio: 0.8; phantom ghost peaks filtering; relative mass defect: 50-7000; the maximum mass difference for feature relationship detection was set to: 0.005 Da and maximum retention time difference: 0.03 min. Feature clustering was performed based on Person’s correlation and features were assumed to be correlated when Person correlation links were applied for a correlation ≥ 0.8 and a p-value significance threshold = 0.05. For the final dataset, which was used for statistical analysis and metabolite identification, two peaks were kept in each cluster for database search, including the most connected and the most abundant feature in the cluster. After the MSCleanR workflow, the final prioritization of features was conducted to ensure high-quality data^37^. Less reliable features were removed based on the coefficient of variation (CV) calculated from drift-corrected samples using QC, filtering out if relative standard deviation (RSD) was greater than 30%. Additionally, features with the diagnostic power (DP) < 2 (defined as the ratio of relative standard deviation across all samples to that across QC samples) were removed^38^.

The data was visualized using MetaboAnalyst 6.0, with log transformation and Pareto-scaling^39^. MS-DIAL and MS-FINDER (v 3.60) were used for peak annotations with a customised compound database. A customised compound mass-spectral database (msp format) in MS-DIAL was created beforehand for containing 735 suspected and earlier identified compounds in similar experiments. Database includes chemical identifiers (*i.e.*, InChIs and InChIKeys), monoisotopic mass, experimental and in-silico mass spectra and other metadata.

**MS-Dial and MSCleanR analysis**

**MS-DIAL parameters for ESI^-^ in Secondary metabolites study**

| Parameter | Unit | Value | Comment |
| --- | --- | --- | --- |
| *Import* | | | |
| Number of files (.abf) per ionisation mode |  |  | 66 files in total:  6 QC samples  4 Blank samples  2 Method blank samples  54 samples |
| Target omics |  | Metabolomics | Suspect Screening list |
| *Data collection* | | | |
| MS1 and MS2 tolerance | Da | 0.01, 0.05 |  |
| Retention time window | min | 1 -20 |  |
| MS range (MS1 and MS2) | Da | 50-1200 |  |
| Maximum charged number |  | 2 |  |
| Consider Cl and Br elements |  | No |  |
| *Peak detection* | | | |
| Minimum peak height | amplitude | 2000 |  |
| Mass slice width | Da | 0.1 |  |
| Smoothing method (smoothing level, minimum peak width) | scan | (3, 5) | Linear weighted moving average |
| *MS2Dec* | | | |
| Sigma window value |  | 0.5 |  |
| MS/MS abundance cut off | amplitude | 100 |  |
| Exclude after precursor ion |  | Yes |  |
| *Identification* | | | |
| Msp file |  | Personalised | Based on excel file (Supporting information) |
| Accurate mass tolerance (MS1) | Da | 0.005 |  |
| Accurate mass tolerance (MS2) | Da | 0.025 |  |
| Identification score cut off | % | 70 |  |
| *Adduct* | | | |
| Molecular species (ESI^-^) |  |  | [M-H]^-^, [M-H_2_O-H]^-^, [M+Na-2H]^-^, [M+Cl]^-^, [M+FA+H]^-^, [M-C_6_H_10_O_4_-H]^-^, [M-C_6_H_10_O_5_-H]^-^, [M-C_6_H_8_O_6_-H]^-^, [2M‑H]^-^, [2M-FA-H]^-^, [M-2H]^2-^ |
| *Alignment* | | | |
| Reference file |  | QC4 | QC sample run in the middle of the sequence |
| Retention time tolerance | min | 0.05 |  |
| MS1 tolerance | Da | 0.005 |  |
| N% detected in at least one group | % | 0 |  |
| Remove features based on blank information |  | Yes |  |
| Sample max/ average blank | fold change | 5 |  |
| Keep ‘reference matched’ metabolite features |  | Yes |  |
| Keep ‘suggested w/o MS2’ metabolite features |  | No |  |
| Keep removable features and assign the tag |  | Yes |  |
| Gap fill by compulsion |  | Yes |  |

**Table S.2. MS-CleanR parameters^*^**

| Parameter | Unit | Value | Comment |
| --- | --- | --- | --- |
| *Before importing to MS-CleanR* | | | |
| Normalisation in MS-DIAL |  |  | LOESS normalisation based on six QC samples in each ESI^+^ and ESI^-^. |
| *Clean MS-DIAL data* | | | |
| Generic filters – What filters to use? |  |  |  |
| Blank ratio |  | 0.8 | Minimum blank ratio |
| Delete ghost peaks? |  | Yes |  |
| Relative standard deviation |  | No |  |
| Incorrect Mass |  | Yes |  |
| Relative Mass Defect | RMD | 50-7000 |  |
| Clustering deltas |  |  |  |
| Maximum mass difference | Da | 0.005 |  |
| Maximum retention time difference | min | 0.03 |  |
| Clustering options |  |  |  |
| Use Pearson correlation to compute clusters  Minimum correlation  Maximum p-value |  | 0.8  0.05 |  |
| *Keep top peak clusters* | | | |
| Selection criteria |  |  |  |
| Intensity (most intense peaks) |  | 2 | Number of peaks to keep (by cluster and by method) |
| Degree (most connected peaks) |  | 2 | As above |

*A tutorial for MS-CleanR is available at: <https://github.com/eMetaboHUB/MS-CleanR/blob/master/MS-Dial-CleanR-Finder-Tutorial-v1c.pdf>
