# Supplementary material for: Yeast–Fusarium interactions and mycotoxin modulation in the phyllosphere
Source: ISME J. 2026 Jul 16;20(1):wrag187. doi: 10.1093/ismejo/wrag187 (PMC13528865; doi:10.1093/ismejo/wrag187)
Supplement: Supplementary file 1 [file supplementary_material_wrag187.zip › Supplementary Figure 1_revised.pdf]

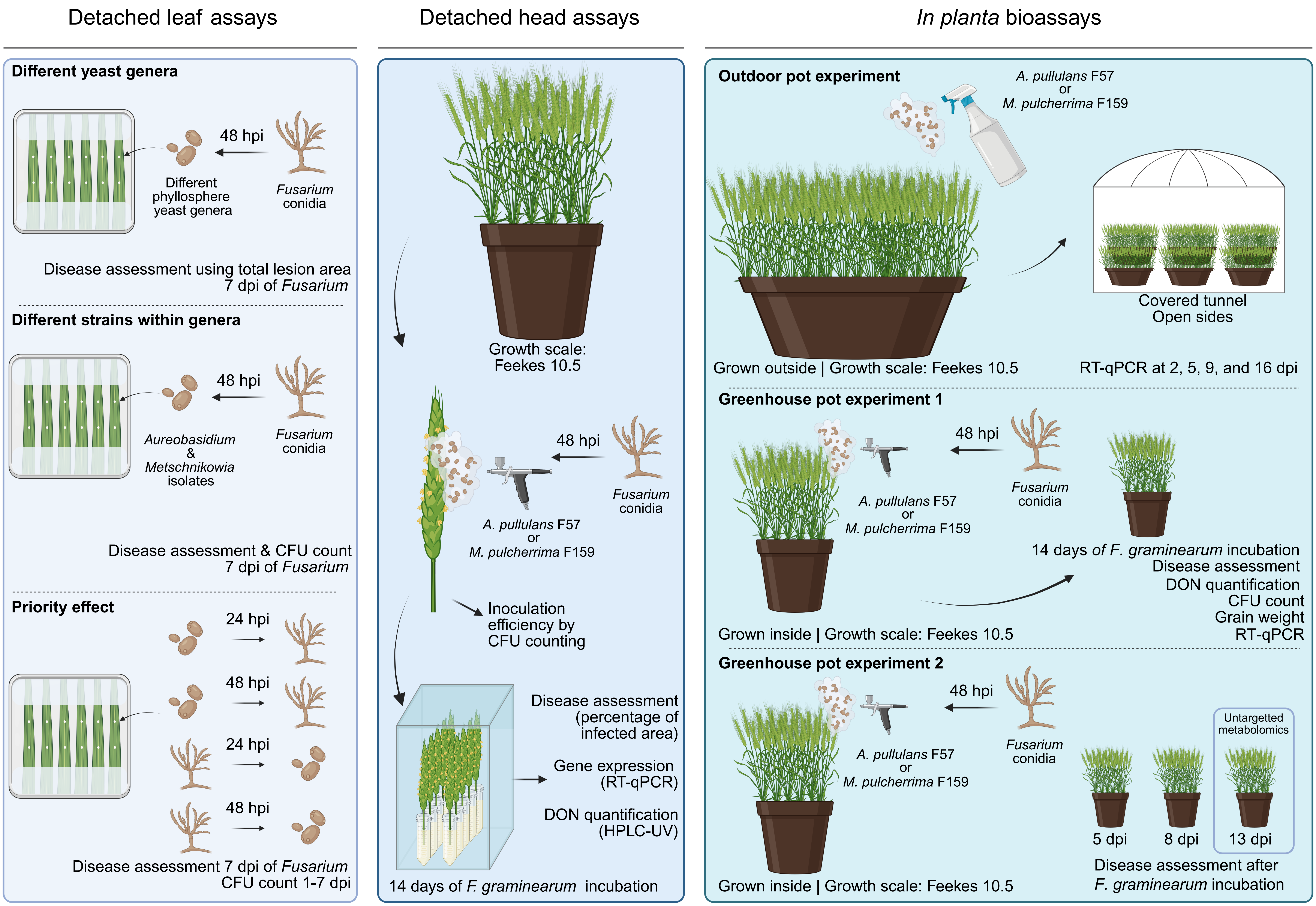
Detached leaf assays
Detached head assays
In planta bioassays
Different yeast genera
48 hpi
Different phyllosphere yeast genera
Fusarium conidia
Disease assessment using total lesion area
7 dpi of Fusarium
Different strains within genera
48 hpi
Aureobasidium & Metschnikowia isolates
Fusarium conidia
Disease assessment & CFU count
7 dpi of Fusarium
Priority effect
24 hpi
48 hpi
24 hpi
48 hpi
Disease assessment 7 dpi of Fusarium
CFU count 1-7 dpi
Growth scale: Feekes 10.5
48 hpi
Fusarium conidia
A. pullulans F57
or
M. pulcherrima F159
Inoculation efficiency by CFU counting
Disease assessment (percentage of infected area)
Gene expression (RT-qPCR)
DON quantification (HPLC-UV)
14 days of F. graminearum incubation
Outdoor pot experiment
A. pullulans F57
or
M. pulcherrima F159
Covered tunnel
Open sides
Grown outside | Growth scale: Feekes 10.5
RT-qPCR at 2, 5, 9, and 16 dpi
Greenhouse pot experiment 1
48 hpi
A. pullulans F57
or
M. pulcherrima F159
Fusarium conidia
14 days of F. graminearum incubation
Disease assessment
DON quantification
CFU count
Grain weight
RT-qPCR
Grown inside | Growth scale: Feekes 10.5
Greenhouse pot experiment 2
48 hpi
A. pullulans F57
or
M. pulcherrima F159
Fusarium conidia
Untargetted metabolomics
5 dpi
8 dpi
13 dpi
Disease assessment after
F. graminearum incubation
Grown inside | Growth scale: Feekes 10.5
