# Supplementary material for: Yeast–Fusarium interactions and mycotoxin modulation in the phyllosphere
Source: ISME J. 2026 Jul 16;20(1):wrag187. doi: 10.1093/ismejo/wrag187 (PMC13528865; doi:10.1093/ismejo/wrag187)
Supplement: Supplementary file 1 [file supplementary_material_wrag187.zip › Supplementary Figure 2_revised.pdf]

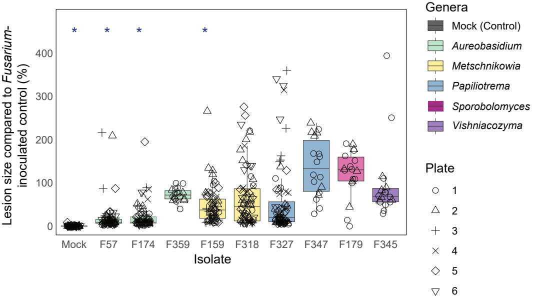

Lesion size compared to *Fusarium*-inoculated control (%)
0
100
200
300
400
*
*
*
*
Mock
F57
F174
F359
F159
F318
F327
F347
F179
F345
Isolate
Genera
Mock (Control)
Aureobasidium
Metschnikowia
Papiliotrema
Sporobolomyces
Vishniacozyma
Plate
1
2
3
4
5
6
