# Supplementary material for: Yeast–Fusarium interactions and mycotoxin modulation in the phyllosphere
Source: ISME J. 2026 Jul 16;20(1):wrag187. doi: 10.1093/ismejo/wrag187 (PMC13528865; doi:10.1093/ismejo/wrag187)
Supplement: Supplementary file 1 [file supplementary_material_wrag187.zip › Supplementary Figure 3.pdf]

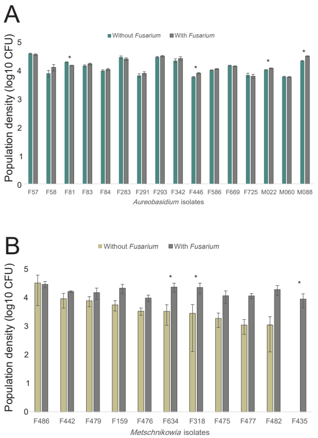
A
Without Fusarium
With Fusarium
Population density (log10 CFU)
5
4
3
2
1
0
F57
F58
F81
F83
F84
F283
F291
F293
F342
F446
F586
F669
F725
M022
M060
M088
Aureobasidium isolates
B
Without Fusarium
With Fusarium
Population density (log10 CFU)
5
4
3
2
1
0
F486
F442
F479
F159
F476
F634
F318
F475
F477
F482
F435
Metschnikowia isolates
