# Supplementary material for: Yeast–Fusarium interactions and mycotoxin modulation in the phyllosphere
Source: ISME J. 2026 Jul 16;20(1):wrag187. doi: 10.1093/ismejo/wrag187 (PMC13528865; doi:10.1093/ismejo/wrag187)
Supplement: Supplementary file 1 [file supplementary_material_wrag187.zip › Supplementary Figure 4.pdf]

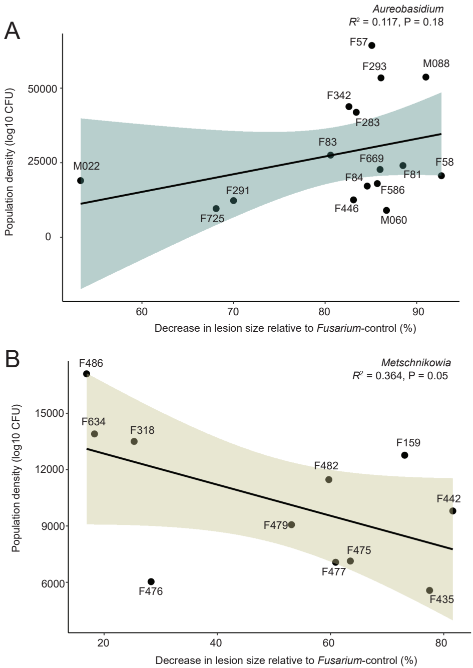
A
Aureobasidium
R^2 = 0.117, P = 0.18
Population density (log10 CFU)
50000
25000
0
60
70
80
90
Decrease in lesion size relative to Fusarium-control (%)
F57
F293
M088
F342
F283
F83
M022
F669
F58
F81
F84
F586
F291
F446
F725
M060
B
Metschnikowia
R^2 = 0.364, P = 0.05
Population density (log10 CFU)
15000
12000
9000
6000
20
40
60
80
Decrease in lesion size relative to Fusarium-control (%)
F486
F634
F318
F159
F482
F442
F479
F475
F477
F476
F435
