# Supplementary material for: Yeast–Fusarium interactions and mycotoxin modulation in the phyllosphere
Source: ISME J. 2026 Jul 16;20(1):wrag187. doi: 10.1093/ismejo/wrag187 (PMC13528865; doi:10.1093/ismejo/wrag187)
Supplement: Supplementary file 1 [file supplementary_material_wrag187.zip › Supplementary Figure 5_revised.pdf]

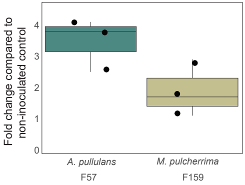
Fold change compared to non-inoculated control
0
1
2
3
4
A. pullulans
F57
M. pulcherrima
F159
