# Supplementary material for: Yeast–Fusarium interactions and mycotoxin modulation in the phyllosphere
Source: ISME J. 2026 Jul 16;20(1):wrag187. doi: 10.1093/ismejo/wrag187 (PMC13528865; doi:10.1093/ismejo/wrag187)
Supplement: Supplementary file 1 [file supplementary_material_wrag187.zip › Supplementary Figure 6_revised.pdf]

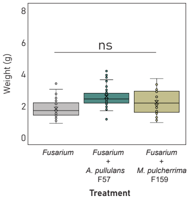

8
6
4
2
0
Weight (g)
ns
Fusarium
Fusarium + A. pullulans F57
Fusarium + M. pulcherrima F159
Treatment
