# Supplementary material for: Yeast–Fusarium interactions and mycotoxin modulation in the phyllosphere
Source: ISME J. 2026 Jul 16;20(1):wrag187. doi: 10.1093/ismejo/wrag187 (PMC13528865; doi:10.1093/ismejo/wrag187)
Supplement: Supplementary file 1 [file supplementary_material_wrag187.zip › Supplementary Figure 7_revised.pdf]

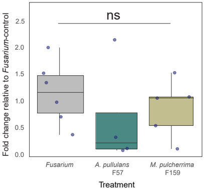

ns
Fold change relative to Fusarium-control
2.5
2.0
1.5
1.0
0.5
0.0
Fusarium
A. pullulans F57
M. pulcherrima F159
Treatment
