# Supplementary material for: Yeast–Fusarium interactions and mycotoxin modulation in the phyllosphere
Source: ISME J. 2026 Jul 16;20(1):wrag187. doi: 10.1093/ismejo/wrag187 (PMC13528865; doi:10.1093/ismejo/wrag187)
Supplement: Supplementary file 1 [file supplementary_material_wrag187.zip › Supplementary Figure and Table legends.docx]

**Supplementary Figure 1.** Graphical illustration of experimental designs used in this study.

**Supplementary Figure 2.** Inhibition of *Fusarium graminearum* in detached leaf assay. Percentage (mean ± SE) of suppressiveness against *F. graminearum* for nine yeast isolates. Fungal growth was quantified using ImageJ software and compared to the growth of the *Fusarium* control. Sample sizes were as follows: n = 5 for the control (mock inoculation with 0.9% NaCl solution supplemented with Tween20), *F. graminearum*, F57, F159, F174, F318, F327 treatments, with each plate containing 6 leaves and 2 lesions per leaf. For the F359, F347, F179 and F345 isolates, n = 3, with each plate consisting of 3 leaves and 2 lesions per leaf. Different symbols indicate origin (plate) of each individual lesion. Boxplots are colored based on genera. A linear mixed-effects model was used to account for the nested experimental design, with yeast treatment as fixed effect, and leaf nested within plate as random effects. Statistical differences between treatments and *Fusarium*-inoculated control were analyzed using Dunnett’s correction on the fitted model, and indicated by an asterisk (*P* < 0.05).

**Supplementary Figure 3.** Population density of *Aureobasidium* and *Metschnikowia* in the presence and absence of *F. graminearum*. All **A)** *Aureobasidium* and **B)** *Metschnikowia* isolates were screened for their ability to colonize the leaf environment in the presence and absence of *F. graminearum* (n = 3, mean ± SE, log10 transformed). Colony forming units were counted after 9 days post inoculation. Statistical analysis was conducted with student t-test for all *Aureobasidium* and *Metschnikowia* isolates, except for *Metschnikowia* isolate F435, for which a Welch two sample t-test was used. Statistical differences are indicated by an asterisk (*P* < 0.05).

**Supplementary Figure 4.** Correlation between disease control (decrease in lesion size relative to *Fusarium*-control) and population density of *Aureobasidium* spp. and *Metschnikowia* spp. (CFU/0.5cm^2^ lesion, log10 transformed). Linear regression analysis was performed for **A)** *Aureobasidium* and **B)** *Metschnikowia* isolates.

**Supplementary Figure 5.** Colonization of phyllosphere yeasts on detached wheat heads. Wheat heads were spray-inoculated at the flowering stage with *A.* *pullulans* F57 and *M. pulcherrima* F159 (n = 3).

**Supplementary Figure 6**. Fresh weight measurements after Fusarium Head Blight (FHB) symptoms (Mean ± SE). Fresh weight measurements were taken to investigate the ability of yeast-treatment to mitigate shivered and empty grains caused by *F.* *graminearum*. Sample sizes were as follows: *F. graminearum* (n = 46), *F. graminearum* + F57 (n = 51), *F. graminearum* + F159 (n = 20). Statistical differences were determined by Mann-Whitney U test (*P* < 0.05).

**Supplementary Figure 7**. *TRI6* gene expression in control (*Fusarium*) wheat heads and those treated with *A.* *pullulans* F57 and *M. pulcherrima* F159 (n_Fusarium_ = 6, n_F57_ = 4, n_F159_ = 5), statistical differences were tested with Kruskal-Wallis (*P* < 0.05). Dots in boxplot indicate individual data points.

**Supplementary Table 1**. Yeast isolates used in this study, indicating isolate ID, genera, species, and source. Species identification was based on ITS rRNA gene sequencing.

**Supplementary Table 2.** Untargeted metabolomic analysis of greenhouse pot experiment 2, including MSI identification level.

**Supplementary Table 3.** Metabolic changes upon yeast and *Fusarium graminearum* inoculation after 13 days. Significantly different compounds detected between: (1) non-inoculated wheat (control) and *A. pullulans* F57-inoculated wheat, (2) non-inoculated wheat and *M. pulcherrima* F159-inoculated wheat, (3) non-inoculated wheat and *Fusarium*-inoculated wheat, and (4) *Fusarium* + *A. pullulans* F57-inoculated and *Fusarium* + *M. pulcherrima* F159-inoculated wheat (One-way ANOVA, Tukey HSD, P < 0.05).

**Supplementary Table 4.** Preliminary genome mining of *A.* *pullulans* F57 and *M. pulcherrima* F159. Hits represent the presence of *TRI12* MFS transporters and a *Sts1/Cut8* complex.
