# Supplementary material for: Yeast–Fusarium interactions and mycotoxin modulation in the phyllosphere
Source: ISME J. 2026 Jul 16;20(1):wrag187. doi: 10.1093/ismejo/wrag187 (PMC13528865; doi:10.1093/ismejo/wrag187)
Supplement: Supplementary file 1 [file supplementary_material_wrag187.zip › Alt.docx]

Alt-text_Supplementary Figure 1: Schematic diagram summarizing the experimental designs used in the study. The graphic shows the experimental set-up of detached leaf assays, detached head assays, outdoor and greenhouse pot experiments, and which corresponding analysis was performed. Arrows indicate timelines, inoculation steps with *Fusarium graminearum* and yeast isolates, and sampling points for colony counts, disease scoring, biomass quantification (RT-qPCR), toxin analysis (HPLC-UV), and metabolomics (LC-MS/MS).

Alt-text_Supplementary Figure 2: Boxplots showing inhibition of *Fusarium graminearum* lesion growth in a detached leaf assay by nine yeast isolates from five different genera. Each boxplot represents percentage suppressiveness relative to the *Fusarium*-inoculated control, with individual lesion measurements overlaid as points using different symbols to indicate plate origin. Box colors correspond to yeast genera, and sample sizes differ by isolate. Inoculation with *Aureobasidium* sp. F57, F174, or *M. pulcherrima* F159 reduces lesion size compared with the *Fusarium*-inoculated control treatment.

Alt-text_Supplementary Figure 3: Figure with two grouped bar charts showing population densities of *Aureobasidium* and *Metschnikowia* isolates on wheat leaves with and without *Fusarium graminearum*. Panel A depicts *Aureobasidium* isolates and panel B depicts *Metschnikowia* isolates. Bars represent log10-transformed colony-forming units per lesion. *Aureobasidium* isolates F446, M022, and M088 show significant increase in colonization capacity compared to the *Fusarium* co-inoculation, while a decrease in population density is observed for F81. *Metschnikowia* isolates F634, F318, and F435 show significant increase in colonization capacity compared to the *Fusarium* co-inoculation.

Alt-text_Supplementary Figure 4: Two scatter plots illustrating the relationship between disease control, measured by lesion size, and yeast population density, based on CFU counts, on wheat leaves. Panel A presents *Aureobasidium* isolates and panel B presents *Metschnikowia* isolates. Each point represents an isolate, with the x-axis showing log10-transformed colony-forming units per lesion and the y-axis showing percentage decrease in lesion size relative to the *Fusarium* control. A fitted linear regression line is displayed in each panel, including the 95% confidence bands.

Alt-text_Supplementary Figure 5: A boxplot showing colonization of wheat heads by *Aureobasidium pullulans* F57 and *Metschnikowia pulcherrima* F159 in a detached wheat head assay. Wheat heads were spray-inoculated at flowering, and yeast population densities are presented for each treatment, based on RT-qPCR.

Alt-text_Supplementary Figure 6: A box plot comparing fresh weight of wheat grain of *Fusarium*-inoculated control versus *A. pullulans* F57 or *M. pulcherrima* co-inoculated treatment groups, showing no significant difference in the experimental group.

Alt-text_Supplementary Figure 7: A box plot comparing the expression levels of Gene TRI6 in *Fusarium*-inoculated control versus *A. pullulans* F57 or *M. pulcherrima*-inoculated treatment groups, showing no significant difference in the experimental group.
